# Dual‐Targeting Peptide‐Guided Approach for Precision Delivery and Cancer Monitoring by Using a Safe Upconversion Nanoplatform

**DOI:** 10.1002/advs.202002919

**Published:** 2021-01-06

**Authors:** Shuai Zha, Ho‐Fai Chau, Wai Yin Chau, Lai Sheung Chan, Jun Lin, Kwok Wai Lo, William Chi‐Shing Cho, Yim Ling Yip, Sai Wah Tsao, Paul J. Farrell, Liang Feng, Jin Ming Di, Ga‐Lai Law, Hong Lok Lung, Ka‐Leung Wong

**Affiliations:** ^1^ Department of Chemistry Hong Kong Baptist University 224 Waterloo Road Kowloon Hong Kong SAR 000000 P. R. China; ^2^ Department of Biology Hong Kong Baptist University 224 Waterloo Road Kowloon Hong Kong SAR 000000 P. R. China; ^3^ State Key Laboratory of Rare Earth Resource Utilization Changchun Institute of Applied Chemistry Chinese Academy of Sciences Changchun 130000 P. R. China; ^4^ Department of Anatomical & Cellular Pathology and State Key Laboratory of Translational Oncology The Chinese University of Hong Kong Kowloon Hong Kong SAR 000000 P. R. China; ^5^ Department of Clinical Oncology Queen Elizabeth Hospital Kowloon Hong Kong SAR 000000 P. R. China; ^6^ School of Biomedical Sciences The University of Hong Kong Kowloon Hong Kong SAR 000000 P. R. China; ^7^ Section of Virology Imperial College Faculty of Medicine Norfolk Place London W12 0BZ UK; ^8^ Department of Applied Biology and Chemical Technology The Hong Kong Polytechnic University Hung Hom Hong Kong SAR 000000 P. R. China; ^9^ Department of Urology The Third Affiliated Hospital of Sun Yat‐sen University 600# Tianhe Road Guangzhou 510630 P. R. China

**Keywords:** EBV‐associated cancer imaging, lanthanide upconversion nanoplatform, peptide‐guided precision cancer therapy, pH‐responsive peptide, selective EBV‐infected cytotoxicity

## Abstract

Using Epstein‐Barr virus (EBV)‐induced cancer cells and HeLa cells as a comparative study model, a novel and safe dual‐EBV‐oncoproteins‐targeting pH‐responsive peptide engineering, coating, and guiding approach to achieve precision targeting and treatment strategy against EBV‐associated cancers is introduced. Individual functional peptide sequences that specifically bind to two overexpressed EBV‐specific oncoproteins, EBNA1 (a latent cellular protein) and LMP1 (a transmembrane protein), are engineered in three different ways and incorporated with a pH‐sensitive tumor microenvironment (TME)‐cleavable linker onto the upconversion nanoparticles (UCNP) NaGdF_4_:Yb^3+^, Er^3+^@NaGdF_4_ (UCNP‐P*_n_*, *n* = 5, 6, and 7). A synergistic combination of the transmembrane LMP1 targeting ability and the pH responsiveness of UCNP‐P*_n_* is found to give specific cancer differentiation with higher cellular uptake and accumulation in EBV‐infected cells, thus a lower dose is needed and the side effects and health risks from treatment would be greatly reduced. It also gives responsive UC signal enhancement upon targeted dual‐protein binding and shows efficacious EBV cancer inhibition in vitro and in vivo. This is the first example of simultaneous imaging and inhibition of two EBV latent proteins, and serves as a blueprint for next‐generation peptide‐guided precision delivery nanosystem for the safe monitoring and treatment against one specific cancer.

## Graphical abstract

The upconversion nanoplatform with desired responsiveness toward tumor microenvironment is introduced. Thereafter, EBV‐cancer model is used to investigate by utilizing the safe nanoplatform *via* targeting LMP1 and EBNA1 with responsive emission, satisfactory prohibitive performances are observed *in vitro* and *in vivo*, thanks to the specific dual‐targeting peptides. This proposed multifunctional nanoplatform can achieve both monitoring and treatment for EBV cancer.

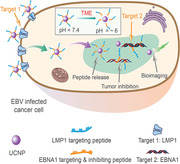

## 1 Introduction

In recent decades, nanomaterials have been researched and investigated as promising agents for tumor bioimaging and cancer treatment in vivo because of their enhanced permeability and retention (EPR) effects.^[^ ^1^, ^2^, ^3^, ^4^ ^]^ Lanthanide‐based upconversion (UC) nanomaterials have emerged as exceptional candidates, superior to molecular compounds, because of their merits of low biotoxicity, high photochemical stability, narrow and sharp emission band, minimal autofluorescence, deep light penetration, and uniform size distribution.^[^ ^5^, ^6^, ^7^, ^8^, ^9^, ^10^, ^11^, ^12^ ^]^ Surface‐functionalized UCNP of uniform quality, morphology and synthetic reproducibility have emerged as an excellent choice in recent studies of nanobiosafety. Although there are numerous laudable examples in applying UC nanomaterials for biological uses,^[^ ^13^, ^14^, ^15^, ^16^, ^17^, ^18^, ^19^, ^20^, ^21^ ^]^ the biomedical application of pH‐responsive peptide‐functionalized stable nanoprobes with responsive UC emission for the inhibition and monitoring of a specific cancer type over normal cells is still rare, and our simultaneous dual‐oncoprotein‐targeting approach for achieving such sought‐after “precision cancer therapy” with UC nanosystems in vivo is novel.

In this work, we adopted Epstein‐Barr virus (EBV) cancer cells as a model to demonstrate the ability of our novel nanoplatform. EBV is a pandemic in human beings and there are ≈200 000 new cancer cases in the world annually.^[^ ^22^, ^23^ ^]^ Notably, EBV is involved in a wide variety of lymphoid and epithelial cancers.^[^ ^24^, ^25^ ^]^ Additionally, EBV causes lifelong latent infection in the host.^[^ ^26^ ^]^ The Epstein‐Barr nuclear antigen 1 (EBNA1), normally in the form of a dimer, plays a significant role in the maintenance, replication, and transcription of the EBV genome and is the only viral latent protein expressed in all EBV‐associated cancers.^[^ ^27^, ^28^, ^29^, ^30^ ^]^ All EBV‐infected cells are EBNA1‐positive. Recently, we have reported four species of dual functional EBNA1‐targeting bioprobes, JLP_2_,^[^ ^31^ ^]^ L_2_P_4_,^[^ ^32^ ^]^ UCNP‐P_4_^[^ ^33^ ^]^ and ZRL_5_P_4_.^[^ ^34^ ^]^ These represent the first generation of bioprobes for concurrent monitoring and inhibition of EBV‐associated cancer cells with responsive emission signal when bound with EBNA1, especially for the UC emission enhancement exhibited in UCNP‐P_4_.

The EBV‐encoded latent infection membrane protein 1 (LMP1) is regarded as one of the primary oncogenes of EBV. LMP1 plays an important role in B cell transformation, proliferation, and survival induced by EBV, induction of epithelial–mesenchymal transition and acquisition of cancer stem cell‐like properties, which are subsequently involved in development and progression of EBV‐associated tumors.^[^ ^35^, ^36^ ^]^ In some EBV associated cancers (for example, EBV lymphomas in immunosuppressed patients), all the tumor cells express LMP1 but only about 40% of nasopharyngeal carcinoma (NPC) patients have LMP1‐positive EBV‐associated tumors.^[^ ^37^, ^38^ ^]^ The reason why only a portion of NPC tumors have LMP1, is that the somatic nuclear factor kappa‐light‐chain‐enhancer of activated B cells (NF‐*κ*B) signaling pathway aberrations have replaced the role of LMP1 in activating the NF‐*κ*B pathway for tumor growth in those NPC tumors^[^ ^39^ ^]^ and this absence of LMP1 expression has an advantage for tumor cells to escape from immune cell recognition.

There are six transmembrane domains (TM 1–6) in the LMP1 structure and the FWLY motif (amino acid, a.a. 38–41) is important for TM1‐2 induction of NF‐*κ*B activation.^[^ ^35^ ^]^ Furthermore, it is well documented that FWLY motif is involved in the constitutive LMP1 aggregation and continuous cytoplasmic C terminus‐mediated signal transduction including the NF‐ĸB signaling pathway which is essential for cell proliferation.^[^ ^36^, ^40^, ^41^, ^42^, ^43^ ^]^

LMP1 is a cell membrane protein, unlike EBNA1 which is a nuclear protein, so LMP1 is likely to be a more accessible drug target, allowing some drug selectivity toward tumor cells. A cell surface or cytoplasmic membrane molecule represents favorable opportunity for cancer cell tracking and may also reduce off‐target effects, as uptaking of a drug into the cell nucleus is not necessary. Hence in theory, the incorporation of tailor‐made peptides could provide selective cytotoxicity to tumor cells with fewer side effects on normal tissues. LMP1 is therefore considered as a new potential target protein to improve the treatment selectivity. The FWLY motif is critical for the mediation of intermolecular interactions with the transmembrane domains of LMP1, so three dual‐EBNA1/LMP1‐targeting protein‐specific peptides (P*_n_*, *n* = 5, 6, and 7) were designed. The previously reported EBNA1‐targeting peptide sequence P_4_ (YFMVF‐GG‐RrRK; YFMVF, EBNA1 binding motif; RrRK, nucleus permeable motif) was combined with the FWLY motif for targeting the membrane LMP1; then the FWLY motif was engineered and placed at different sites: the C‐terminal side (P_5_: ‐Ahx‐YFMVFGGRrRKGGFWLY), (P_6_: ‐Ahx‐RrRKGGYFMVFGGFWLY), or the N‐terminal site (P_7_: ‐Ahx‐FWLYGGRrRKGGYFMVF) to evaluate the effect on killing EBV‐infected cancer cells. Imine linkers with pH‐sensitive behaviors were also introduced between NaGdF_4_:Yb^3+^, Er^3+^@NaGdF_4_ (UCNP) and the dual‐targeting peptides allow the intracellular release of the designed peptides in EBV‐infected cancer cells. This peptide‐guided dual‐targeting nanosystem was engineered to specifically traffic and deliver the peptides in EBV‐associated cancer cells with responsive upconversion emission property. Targeting the tumor cell surface protein plus the differential release of our anti‐EBV protein peptide in the acidic tumor microenvironment helps to minimize undesirable and unintended damage to normal cells.

**Scheme 1:**
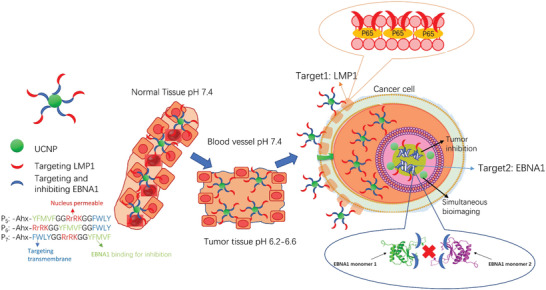
The schematic illustration showing the path of entry of the nanoprobe UCNP‐P*_n_*, into an EBV‐infected cancer cell from normal tissues through sequential and selective targeting.

To assess our novel strategy in delivering precision monitoring and cancer treatment, our new anti‐EBV compounds were tested with various EBV‐ or LMP1‐positive and ‐negative cancer cell models. We have found that the presence of FWLY motif located at the C‐terminus (P_5_) of the EBV‐targeting peptide, seems to facilitate the entrance of our anti‐EBV nanodrug into EBV‐infected cells and stronger affinity towards the nuclear proteins for subsequent therapeutic applications. **Scheme** **1** illustrates a predicted in vivo scenario of how the new nanoplatform can be delivered from the tumor microenvironment, enter an EBV‐positive cell via the cell surface LMP1 protein, penetrate into the cell nucleus and target EBNA1. In theory, the whole process could be monitored via responsive UC emission, and the EBV‐positive cell would be killed by disruption of EBNA1 dimerization. Here, we have characterized the newly constructed nanoprobes UCNP‐P*_n_* (*n* = 5, 6, and 7) to show that UCNP‐P_5_ is the most potent pH‐responsive anti‐EBV nanoplatform with responsive upconversion emission, promising in vitro and in vivo antitumor activities and enhanced cellular and tumor uptake. This can provide a blueprint for such dual‐target agents to be translated into therapies for EBV‐associated cancer patients as well as other cancer patients.

## 2 Results and Discussion

### 2.1 Synthesis and Characterization

The transmission electron microscopy (TEM) images of NaGdF_4_: Yb^3+^, Er^3+^ (size: ≈25 nm), NaGdF_4_: Yb^3+^, Er^3+^@NaGdF_4_ (size: ≈30 nm) and UCNP‐P_5_ (size: ≈33 nm) are shown in Figure S1a–c (Supporting Information), respectively. As seen in Figure S1c (Supporting Information), a thin layer on the surface of UCNP can be observed in the inset. Similar TEM images of UCNP‐P_6_ and UCNP‐P_7_ are shown in Figures S2 and S3 (Supporting Information). A size increase of around 3 nm in UCNP‐P*_n_* (*n* = 5, 6, and 7) compared to UCNP was observed, implying successful bioconjugation of UCNP with P*_n_* (*n* = 5, 6, and 7). Likewise, the old nanoprobe UCNP‐P_4_,^[^ ^33^ ^]^ which targets EBNA1, was also synthesized with uniform morphology, as shown in Figure S4 (Supporting Information). UCNP‐P_4_ was included as a control compound.

To further investigate the crystal phase of UCNP and UCNP‐P*_n_* (*n* = 5, 6, and 7), the X‐ray diffraction (XRD) patterns of the as‐prepared samples were obtained. As shown in Figure S5 (Supporting Information), the XRD patterns of UCNP‐P*_n_* (*n* = 5, 6, and 7) were consistent with UCNP in terms of the diffraction angles the patterns of UCNP and UCNP‐P*_n_* (*n* = 5, 6, and 7) and matched well with the standard hexagonal phase structure of NaGdF_4_ (ICDD#27‐0699), which indicates that there is no impure phase introduced during the process of surface modification. All the samples formed the pure hexagonal phase structure.

The corresponding Fourier‐transform infrared (FTIR) transmission spectrum was obtained to study the process of peptide conjugation with UCNP. As shown in Figure S6 (Supporting Information), the stretching vibration of the C—H bond showed absorption peaks at 2928 and 2856 cm^−1^.^[^ ^44^ ^]^ After the coating of dual‐targeting protein‐specific peptides, the stretching vibration of the aromatic C—C bond (1622, 1515 cm^−1^) and C—H bond (837, 801 cm^−1^) is observed as doublet absorption peaks because of the existence of benzene ring in the FWLY motif.^[^ ^45^ ^]^ Therefore, the surface modification was evidenced and confirmed by the corresponding FTIR transmission spectrum. Additionally, the energy‐dispersive spectroscopy (EDS) spectra of UCNP and UCNP‐P*_n_* (*n* = 5, 6, and 7) in Figure S7 (Supporting Information) further manifested the elemental composition of nanoprobes.

To further evaluate the aggregation degree of the as‐prepared samples, dynamic light scattering (DLS) measurements and stability studies were carried out (Figure S8, Supporting Information). The solutions of UCNP and UCNP‐P*_n_* (*n* = 5, 6, and 7) displayed negligible aggregation in phosphate buffer solution (PBS) and fetal bovine serum (FBS) after 1, 4, and 7 d, revealing desirable stability, low aggregation tendency and uniform size distribution, which are highly consistent with TEM results.

**Figure 1:**
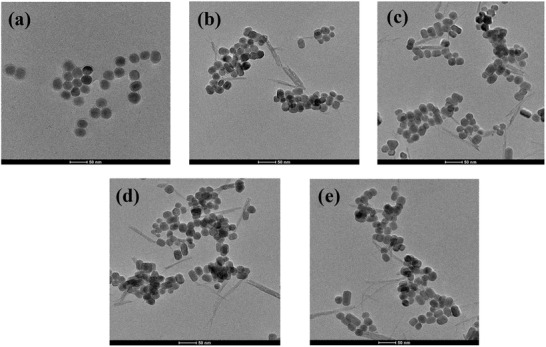
TEM images of UCNP‐P_5_ at a) pH 7.0 b) pH 6.5 c) pH 6.0 d) pH 5.5 e) pH 5.0 PBS buffer.

The stability and capability of cleavage of the pH linker were also studied. The UV–vis absorption spectra of UCNP‐P_4_ and UCNP‐P*_n_* (*n* = 5, 6, and 7) under different time intervals (Conc.: 0.5 mg mL^−1^) were recorded as shown in Figure S9 (Supporting Information). The characteristic peak of the aromatic group was observed in the range of 260–280 nm^[^ ^46^ ^]^ and tiny differences of absorbance peak intensity were observed among all nanoprobes during the experimental period of 7 d, which were quantified and summarized in Table S1 (Supporting Information) for each time interval. These indicated that the peptide layer on the nanoprobes also had excellent stability as the nanoprobes provided solid support for the peptide coating layer. More importantly, TEM images of UCNP‐P_5_ at different pH buffers were observed and investigated in **Figure** **1**, providing direct evidence on pH‐responsive peptide release from UCNP. Moreover, remarkable differences in absorbance intensity were observed in the range of pH 7.4 to pH 5.0 buffers, as shown in Figure S10 (Supporting Information).

Additionally, quantitative analysis of peptide release behaviors of UCNP‐P*_n_* (*n* = 5, 6, and 7) showed that over 80% of specific peptides (P_5_, P_6,_ and P_7_) were released from the initial nanoprobes as the pH decreased, as displayed in Figure S10 and Table S2 (Supporting Information). It is worth noting that the sizes of nanoprobes UCNP‐P*_n_* (*n* = 5, 6, and 7) when measured with DLS decreased significantly in buffers of pH 7.4 to pH 5.0 whereas the size of UCNP remains nearly the same in different pH buffers, as shown in Figure S11 (Supporting Information). This suggests the pH‐cleavable linker, introduced in the nanoprobes by a Schiff base reaction, has an excellent sensitivity towards an acidic environment. The nanoprobes could be separated into two parts, UCNP and P*_n_* (*n* = 5, 6, and 7), upon inducing the cleavage of the pH imine linker in a weakly acidic environment (mimicking tumor microenvironment). Hence, it is likely that the nanoprobes are pH‐responsive, which is consistent with our expectation. The surface charge of the samples was also recorded by zeta‐potential measurements in Figure S12 (Supporting Information). The results showed that the initially negatively charged surface of UCNP became positive due to the positively charged amino group from the three candidate peptides, indicating the successful conjugation of dual‐targeting peptides.

To further confirm that the peptides were intact upon their pH‐induced release from UCNP, both analytical HPLC and ESI‐MS were conducted. HPLC showed no difference in the retention times between original peptides and the solution released from corresponding nanoprobes. ESI‐MS also confirmed both pre‐conjugated and post‐release samples are the desired peptides (Figures S13–S15, Supporting Information). Therefore, these results indicate that the peptides can be released intact from the nanoprobes under acidic conditions.

### 2.2 Photophysical Properties

**Figure 2:**
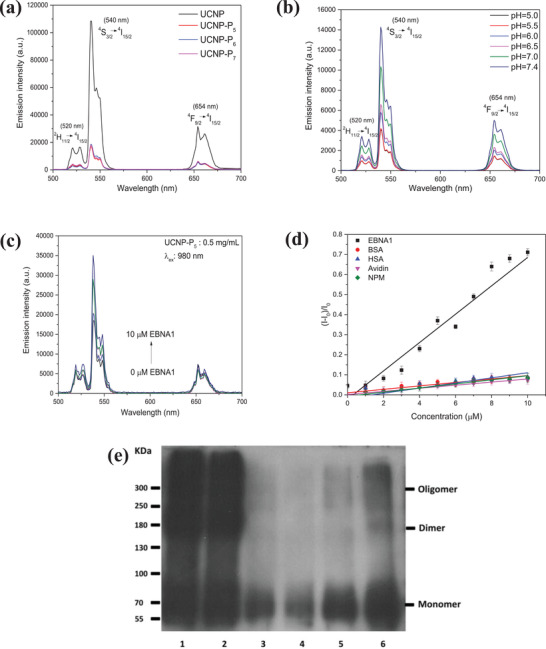
a) The upconversion visible emission spectra of UCNP and UCNP‐P*_n_* (*n* = 5, 6, and 7) b) pH‐responsive upconversion visible emission spectrum of UCNP‐P_5_. c) Luminescence titration of UCNP‐P_5_ (conc.: 0.5 mg mL^−1^; excitation at 980 nm) towards EBNA1; d) change in emission intensity of UCNP‐P_5_ on addition of EBNA1, BSA, HSA, Avidin and NPM; e) EBNA1 dimerization inhibition assay on 1: PBS, 2: UCNP, 3: UCNP‐P_4_, 4: UCNP‐P_5_, 5: UCNP‐P_6_ and 6: UCNP‐P_7_.

The upconversion luminescence properties of the UCNP and UCNP‐P*_n_* (*n* = 5, 6, and 7) were evaluated in DI‐water at the same concentration of 0.5 mg mL^−1^ under continuous‐wave 980 nm near‐infrared laser excitation. All of the as‐prepared samples exhibited three characteristic upconversion emission bands centered at the wavelength of 520, 540, and 654 nm, which correspond to the ^2^H_11/2_, ^4^S_3/2_→^4^I_15/2,_ and ^4^F_9/2_→^4^I_15/2_ transitions respectively. These represent efficient energy transfer from trivalent ytterbium ion (Yb^3+^) to trivalent erbium ion (Er^3+^), as shown in **Figure** **2**a. The upconversion emission intensity of UCNP is approximately sixfold higher than that of UCNP‐P*_n_* (*n* = 5, 6, and 7), this is attributed to the quenching effect from the peptide coating.

To further verify the association between the pH‐responsive property of the nanoprobes in aqueous solution and their optical properties, the upconversion visible spectra of UCNP‐P_4_, UCNP, and UCNP‐P*_n_* (*n* = 5, 6, and 7) were systematically investigated, as shown in Figure 2b and Figure S18 (Supporting Information), respectively. It is noted that UCNP‐P_4_ and UCNP‐P*_n_* (*n* = 5, 6, and 7) showed responsive upconversion emission and a twofold enhancement was observed when the environment was adjusted from pH 7.4 to pH 5.0. For comparison, UCNP showed negligible difference in its upconversion emission intensities in different pH buffers. The reduction of the quenching effect facilitates the emission intensity recovery of peptide capped nanoprobes. This implies that the cleavage ability of the pH linker in acidic environment of the nanoprobes is highly sensitive. The outstanding pH‐responsiveness demonstrated in fluorescent studies is consistent with the results of TEM images of nanoprobes at different pH buffers.

### 2.3 Luminescence Titration Assays

Luminescence titration assays in PBS buffer were conducted with five proteins: BSA, HSA, avidin, NPM, and EBNA1 to evaluate the binding affinity of UCNP and UCNP‐P*_n_* (*n* = 5, 6, and 7). As shown in Figure S19 (Supporting Information), there was nearly no emission difference in UCNP upon the addition of these five proteins; the same was observed for UCNP‐P*_n_* (*n* = 5, 6, and 7) with the addition of NPM, avidin, HSA and BSA (Figures S20–S22, Supporting Information, respectively). A twofold enhancement in emission intensity was observed for UCNP‐P*_n_* (*n* = 5, 6, and 7) upon addition of 10 × 10^−6^ m EBNA1 when compared with the emission of UCNP‐P*_n_* (*n* = 5, 6, and 7) without any additives (Figure 2c, Figures S20c and S22a, Supporting Information). Additionally, the changes in emission intensity of UCNP and UCNP‐P_n_ (*n* = 5, 6, and 7) toward EBNA1, BSA, HSA, avidin and NPM are plotted in Figure S19f (Supporting Information), Figure 2d, Figures S20f and S22f (Supporting Information), respectively. The results implied that the YFMVF motif (for targeting EBNA1) of the newly designed peptides on the UCNP surface could bind with EBNA1 and induce aggregation between the nanoprobes and EBNA1,^[^ ^33^ ^]^ hence leading to the emission enhancement. It also indicated that UCNP‐P*_n_* (*n* = 5, 6, and 7) is highly selective to EBNA1 over NPM, avidin, BSA and HSA, which is consistent with our previous work on UCNP‐P_4_. The results were also supported by molecular dynamics (MD) simulations, which showed that stable RMSD values were obtained for all three peptides binding to the dimerization surface (Figures S16 and S17, Supporting Information).

### 2.4 EBNA1 Dimerization/Oligomerization Inhibition Assays

EBNA1 dimerization/oligomerization inhibition assays were performed to reveal the ability of interrupting EBNA1 dimerization/oligomerization in the presence of the previous nanoprobe UCNP‐P_4_ and the nanoplatforms in situ. As shown in Figure 2e, UCNP‐P_4_ and UCNP‐P*_n_* (*n* = 5, 6, and 7) could hinder EBNA1 dimerization/oligomerization at a low concentration of 0.3 µg µL^−1^, UCNP‐P_4_, UCNP‐P_5,_ and UCNP‐P_6_ are able to eradicate all 90 µg EBNA1 dimers and oligomers, showing the strongest inhibitory capacity among all the samples.

The density of EBNA1 dimers was maintained at the same high level after incubation with PBS buffer and UCNP. These results are consistent with the data from luminescence titration assay.

### 2.5 Cellular Distribution in EBV‐Infected Cells

**Figure 3:**
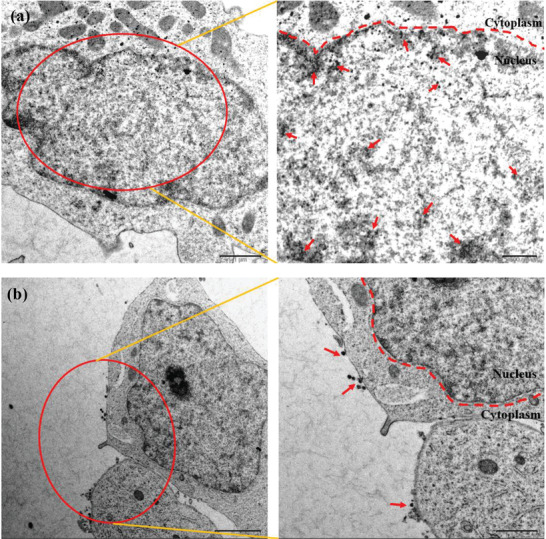
Bio‐TEM images of the localization of a) UCNP‐P_5_ and b) UCNP in EBV‐infected C666 cells after 24 h treatment.

To further evaluate the cellular distribution of the nanoplatforms, bio‐TEM analysis was carried out in EBV‐infected C666 cells. The cells were harvested, fixed and sectioned for bio‐TEM after coincubation with UCNP and UCNP‐P_5_ for 24 h. UCNP‐P_5_ was mainly located in nucleus, but some was in the cytoplasm, as shown in **Figure** **3**a. The dual‐targeting peptide P_5_ may endow the initial UCNP with the ability of entering EBV‐infected C666 cell nucleus due to its RrRK motif. In sharp contrast, as shown in Figure 3b, UCNP was located in the cell membrane and cytoplasm, even the majority of them was adjacent to cell membrane and none of them arrived at the nucleus.

### 2.6 Cellular Uptake ICP‐MS

**Figure 4:**
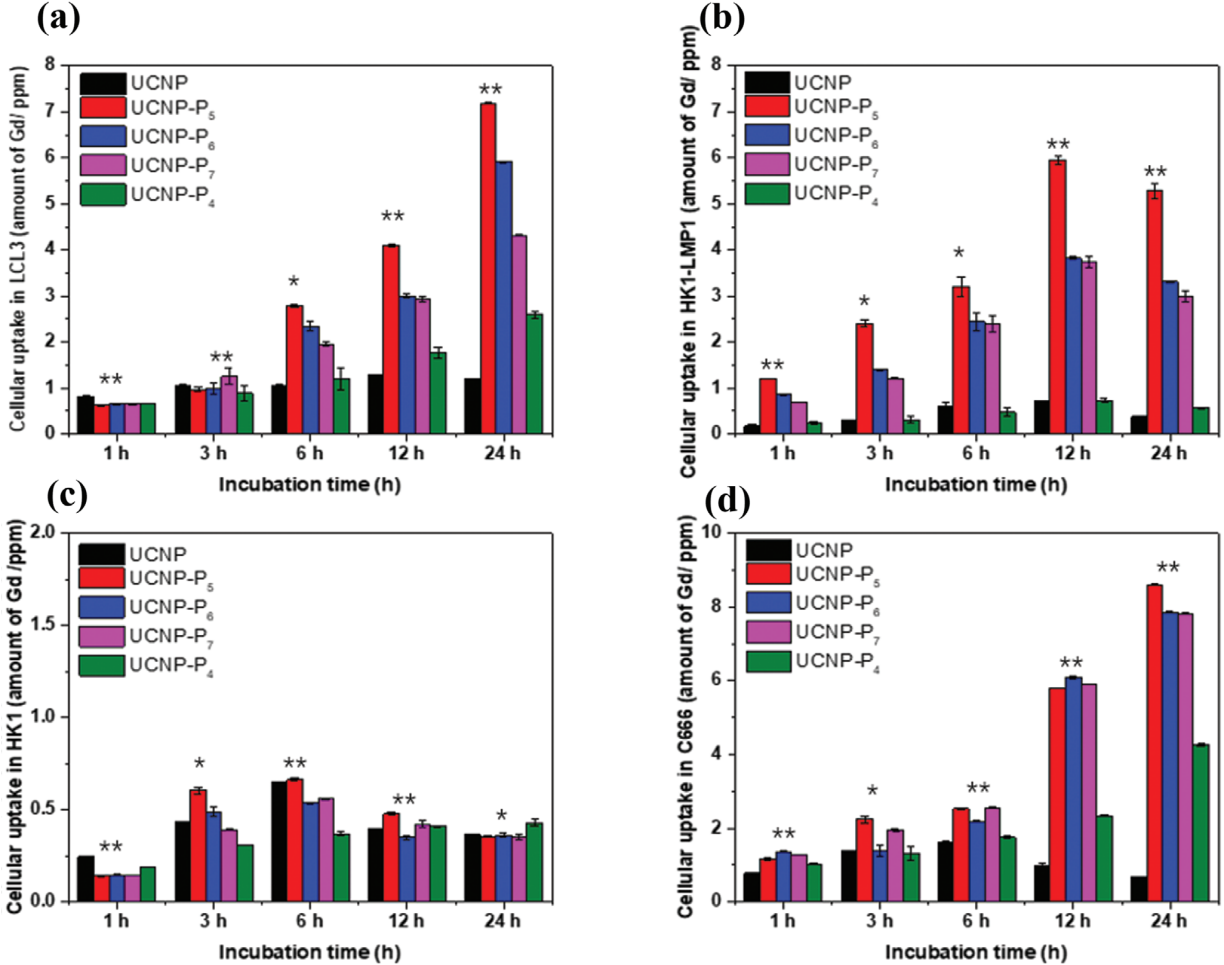
The cellular uptake profile of UCNP, UCNP‐P*_n_* (*n* = 5, 6, and 7) and UCNP‐P_4_ by ICP‐MS in a) LCL3, b) HK1‐LMP1, c) HK1, d) C666. Data are presented as mean ± standard deviation (SD), *n* = 3, statistically significant differences between various treatments are calculated by Student's *t*‐test (*P < 0.05, **P < 0.01).

Bio‐TEM results demonstrated that nanoplatforms were capable of entering the nuclei of EBV‐associated tumor cells. Subsequently, the quantitative distribution and uptake efficiency of nanoprobes in vitro in C666, HK1, HK1‐LMP1, and LCL3 cells lines were monitored and investigated by evaluating the lanthanide content originating from the nanoprobes using inductively coupled plasma mass spectrometry (ICP‐MS). As shown in **Figure** **4**, UCNP‐P_5_ exhibited the largest amount of cellular uptake in LCL3, HK‐LMP1, and C666 cells. UCNP‐P_6_ and UCNP‐P_7_ showed similar uptake rate among these cell lines due to enhanced cell permeability with hydrophilic peptides and effective peptide‐protein binding, indicating interaction between the specific peptides and LMP1 and EBNA1. In addition, the Gd amounts of UCNP‐P_5_ is over twice the amount of old nanoprobes UCNP‐P_4_ in LCL3 and C666 cell lines after 24 h incubation time, which demonstrated that the new generation of nanoprobes exhibited the extraordinary high cellular uptake and accumulation inside EBNA1‐positive cells and LMP1‐positive cells compared with UCNP‐P_4_ thanks to the dual‐EBNA1/LMP1‐targeting ability. For the uptake analysis using the HK1 and HK1‐LMP1 cell pair, the uptake of UCNP‐P_5_ was approximately twofold higher than UCNP‐P_6_ and UCNP‐P_7_, while negligible low signals were observed for all nanocompounds in HK1 cells. This indicated that the location of the C‐terminal FWLY motif is critical for the uptake of UCNP‐P_5_, and its uptake is LMP1‐dependent. The Gd uptake amount in UCNP among all the cell lines is much lower than other nanoprobes because there are no specific peptides coated on the UCNP, hence, UCNP cannot attach to and accumulate in EBV‐positive or LMP1‐positive cells selectively.

### 2.7 In Vitro Toxicity Study

The inhibitory effects of the novel nanoprobes on both EBV‐infected cancer cells and non‐EBV‐infected cancer cells were investigated by MTT assays to test the cell viability. The cytotoxic assays were performed in EBV‐positive C666 and NPC43 cells, LMP1‐positive LCL3 and Raji cells, and the LMP1‐positive and ‐negative HK1 cell pair. HK1, MRC‐5 and HeLa cell lines are EBV‐negative and they serve as negative controls. All the cell lines applied in this study are listed in Table S3 (Supporting Information). The MTT cell viability assays of P*_n_* (*n* = 5, 6, and 7) were first performed, as shown in Figures S23 and S24 (Supporting Information). The results revealed high cytotoxicity among EBV‐positive cells (C666, and NPC43) and LMP1‐positive cells (LCL3) when compared with EBV‐negative cell lines (MRC‐5, HeLa, HK1). In addition, UCNP‐P_4_, EBNA1‐specific peptide coated UCNPs was included as control group, so the ability of inhibiting EBV‐related cancer cells can be directly compared with UCNP‐P*_n_* (*n* = 5, 6, and 7). As shown in Figures S25 and S26 (Supporting Information), UCNP‐P_6_ exhibits the strongest inhibitory effect in all EBV‐positive cells and LMP1‐positive cells after 24 h incubation except the HK1‐LMP1 cell line. This might be due to the fact that the recipient HK1 cells are not dependent on the LMP1‐induced NF‐*κ*B signal for cell growth, a property selected as its EBV genome was previously lost during long‐term culture. P*_n_* (*n* = 5, 6, and 7) did not cause any significant cytotoxicity in the EBV‐negative cell lines (HK1, MRC‐5 and HeLa) even at a high dosage of 200 µg mL^−1^. In addition, the IC_50_ values of UCNP‐P_5_ in EBV‐positive cell lines C666 and NPC43 (C666, IC_50_ = 37 µg mL^−1^; NPC43, IC_50_ = 38 µg mL^−1^;), LMP1‐positive LCL3 (IC_50_ = 19 µg mL^−1^) are much lower than those in EBV‐ and LMP1‐negative HK1 cells (IC_50_ = 1027 µg mL^−1^), EBV‐negative HeLa (IC_50_ = 1086 µg mL^−1^) and MRC‐5 (IC_50_ = 1280 µg mL^−1^) cells. Similar results were observed with UCNP‐P_5_ and UCNP‐P_7_, demonstrating the selectivity of the newly designed dual‐targeting protein‐specific peptides on UCNP‐P*_n_* (*n* = 5, 6, and 7) and pH‐responsive linkers which facilitate the delivery of the peptides toward EBV‐associated cancer cell lines, thereby verifying that UCNP‐P*_n_* (*n* = 5, 6, and 7) can selectively kill EBV‐infected carcinoma cells but remain relatively nontoxic to non‐EBV‐infected tumor cells.

The IC_50_ (half inhibitory concentration) values of EBNA1‐positive and LMP1‐positive cells are summarized for each sample in Table S4 (Supporting Information). Notably, these results are consistent with our previously reported probes L_2_P_4_, UCNP‐P_4,_ and ZRL_5_P_4_, which showed significant inhibitory effect on EBV‐infected cell lines. More importantly, the results from molecular dynamics (MD) simulations (Figure S16, Supporting Information) suggested that the binding affinity of P_6_ with EBNA1 is higher than P_5_. However, better uptake in vitro of UCNP‐P_5_ is observed (Figure 4). Therefore, it is possible that UCNP‐P_6_ exhibits a stronger inhibitory effect than UCNP‐P_5_ in the short term (24 h). During a longer experimental period, UCNP‐P_5_ exceeded UCNP‐P_6_ in a 5 d cytotoxicity test and exhibited the best inhibitory performance among EBNA1‐positive and LMP1‐positive cells (Figure S25e, Supporting Information), which demonstrated that UCNP‐P_5_ would be efficiently taken up and accumulated by EBV‐related cancer cells. It is worth noting that UCNP‐P*_n_* (*n* = 5, 6, and 7) is more selective towards EBV‐infected carcinoma cells compared with the previous bioprobes due to the conjugation of the dual‐EBNA1/LMP1‐targeting peptides on the surface of UCNP. In addition, the cytotoxicity of UCNP‐P_5_ was much higher than UCNP‐P_4_ and exhibited lower IC_50_ values as shown in Table S4 (Supporting Information), especially in LMP1‐positive cells, which indicated that UCNP‐P_5_ is likely to be more able to eradicate EBV‐infected tumor cell progression compared with old generation nanoprobe UCNP‐P_4_. The transmembrane protein LMP1 in EBV‐infected cancer cells is first targeted by the FWLY membrane‐targeting motif, then the EBNA1‐inhibiting YFMVF motif and nucleus‐permeable RrRK motif are prone to cause inhibitory effect on the nucleus, where EBNA1 is initially located. Therefore, the dimerization process of EBNA1 can be hindered efficiently and EBV‐infected tumor cells were significantly inhibited. UCNP‐P_5_ shows promising potential for being an anti‐tumor candidate of multi‐targeted therapy and monitoring with pH‐responsive property.

### 2.8 NF‐*κ*B Signaling Analysis by Western Blotting

**Figure 5:**
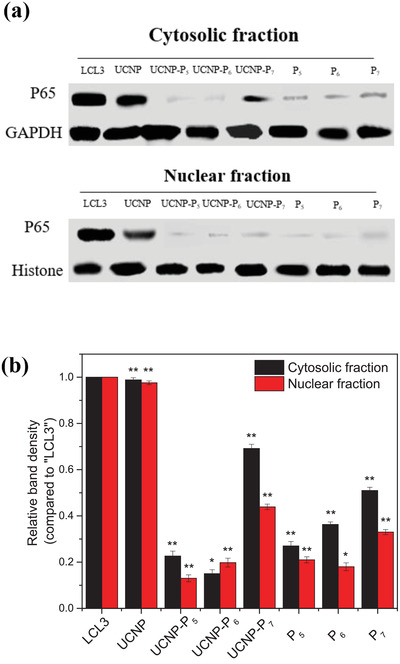
a) Western blotting of UCNP and UCNP‐P*_n_* (*n* = 5, 6, and 7) in cytosolic and nuclear fraction in LCL3 cells. b) Quantitative analysis of p65 protein expression level in cytosolic and nuclear fraction in LCL3 cells after various treatments. *P < 0.05, **P < 0.01.

NF‐ĸB signaling pathway is critical for the survival of EBV‐infected tumor cells. Here we investigated if our nanoprobes could affect this oncogenic pathway. EBV‐positive LCL3 and C666 cells were used as models, EBV‐negative HK1 cells were used as control and Western blot was used to analyze the protein expression of p65, a major transcription factor in the NF‐ĸB family. As shown in **Figure** **5**a and Figure S27a (Supporting Information), UCNP‐P_6_ showed the lowest protein expression level of p65 as a whole, indicating the down‐regulation effect on specific LCL3 and C666 cells, respectively. GAPDH and Histone are regarded as internal standards in cytosolic and nuclear fractions, respectively. In the cytosolic fraction, UCNP‐P_6_ showed the most significant inhibitory effect on p65 (85% and 87% of reduced p65 expression level in LCL3 and C666 cells, respectively) whereas in the nuclear fraction, P_5_ and UCNP‐P_5_ displayed the strongest binding affinity toward p65 with 86% and 89% decreased expression in LCL3 and C666 cells respectively (Figure 5b and Figure S27b, Supporting Information). This is consistent with the cellular uptake results. The negligible differences of p65 expression level in HK1 cells were quantified after various treatments in Figure S28 (Supporting Information).

### 2.9 In Vitro Confocal Imaging

After the study of cellular uptake, fluorescent confocal microscopy was used to study the subcellular localization with the same set of EBV‐/LMP1‐positive and ‐negative cell lines. All these cell lines were treated with as‐prepared samples for different time intervals of 1, 3, 6, 12, and 24 h. Fluorescence signals in the cells were imaged under 980 nm excitation.

**Figure 6:**
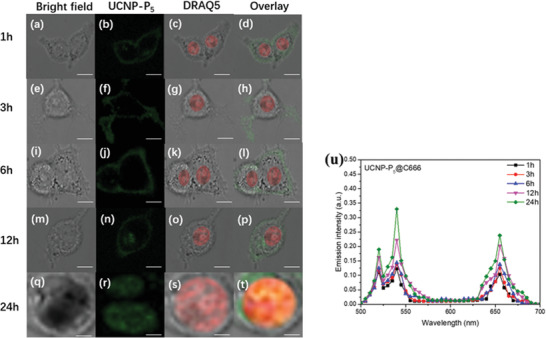
Two‐photon confocal images of UCNP‐P_5_ in EBV‐positive C666 cells (*λ*_ex_ = 980 nm, *λ*_em_ = 500–700 nm); a–d) bright field, UCNP‐P_5_ treated with C666 cells for 1 h, DRAQ5 fluorescence and overlay image respectively; e–h): bright field, UCNP‐P_5_ treated with C666 cells for 3 h, DRAQ5 fluorescence and overlay image respectively; i–l): bright field, UCNP‐P_5_ treated with C666 cells for 6 h, DRAQ5 fluorescence and overlay image respectively; m–p): bright field, UCNP‐P_5_ treated with C666 cells for 12 h, DRAQ5 fluorescence and overlay image respectively; q–t) bright field, UCNP‐P_5_ treated with C666 cells for 24 h, DRAQ5 fluorescence and overlay image respectively; u) Lambda scan of UCNP‐P_5_ in EBV‐positive C666 cells in different time intervals of 1, 3, 6, 12, and 24 h.

As shown in **Figure** **6**, the UCNP‐P_5_ nanoprobe was mainly localized in the cell membrane and weak signals were observed in the cytoplasm in C666 cells from 1 to 3 h. From 6 h onwards, disperse nuclear signal was observed, and only strong signals in the nuclei were observed 24 h. For EBV‐ and LMP1‐negative HK1 cells, as shown in Figure S29 (Supporting Information), very weak signals were detected at the cell membrane from 1 to 6 h, but from 12 h onwards, barely detectable signals were observed, as both targeted proteins, LMP1 and EBNA1, are absent in this cell line. For HK1‐LMP1 cells, weak signals were observed in cell membrane and cytoplasm from 1 to 3 h, increased fluorescent signals with similar patterns were observed from 6 to 24 h (Figure S30, Supporting Information). For LCL3 cells, from 1 to 6 h fluorescent signals were mainly observed in cell membrane, cytoplasmic signals were detected at 12 hours, and weak nuclear signals were observed at 24 hours (Figure S31, Supporting Information).

It is likely that the FWLY motif in UCNP‐P_5_ can enhance the cellular uptake with LMP1 located on the plasma membrane and endoplasmic reticulum, as reflected by the HK1‐LMP1 and HK1 cell pair. For C666 and LCL3 cells, the nanoplatforms are likely to be attracted onto the transmembrane, probably via endocytosis to enter the cytoplasm, and eventually arrive at the nuclei with assistance of the RrRK nuclear location signal sequence, eventually binding with the EBNA1 protein by the YFMVF motif. Lambda scans were run on all the cells and the in vitro emission spectra were recorded at each time interval to detect erbium transitions, since its emission intensity can be regarded as direct evidence of the presence of nanoplatforms. More importantly, a twofold responsive UC emission enhancement was observed after 24 h incubation in EBV‐positive cells, which is consistent with luminescence titration results. Furthermore, the emission was detected mainly from the nucleus, which can be confirmed by fluorescence of DRAQ5 nuclear dye.

The UCNP control was rapidly excreted out of the cells, therefore, UCNP not only had no inhibitory effect on any cells, but also exhibited weak or absent emission signals after 24 h, as shown in Figures S32–S35 (Supporting Information). Moreover, similar to UCNP‐P_5_, UCNP‐P_6_ and UCNP‐P_7_ exhibited twofold responsive emission enhancement mainly from the nuclei in C666 cells as well, while in HK1 cells the intensity declined dramatically, and no signals could be detected. Notably, the emission intensity from cellular transmembrane was maintained even after 24 h incubation in HK1‐LMP1 and LCL3 cells, as shown in Figures S36–S43 (Supporting Information), respectively. In addition, UCNP‐P_4_ only exhibited strong emission in the nucleus of EBNA1‐positive C666 cells after 24 h incubation, as shown in Figure S44 (Supporting Information).

As we had demonstrated previously, the hydroxyl and amine groups with high vibrational energies from the peptide coating would quench the excited state of lanthanide ions.^[^ ^33^ ^]^ Strong interactions of the YFMVF and RrRK motifs with EBNA1 can disturb the surface quenching process and, as a result, the nanoprobes exhibited a recovery in emission signal. Once the nanoplatforms are taken up into a tumor cell, their pH‐responsive linkers are presumably cleaved in the weakly acidic tumor microenvironment in the cancer cell, and more peptides will be released into, hence enhancing the inhibitory activity.

### 2.10 In Vivo Tumor Suppression and Biodistribution

**Figure 7:**
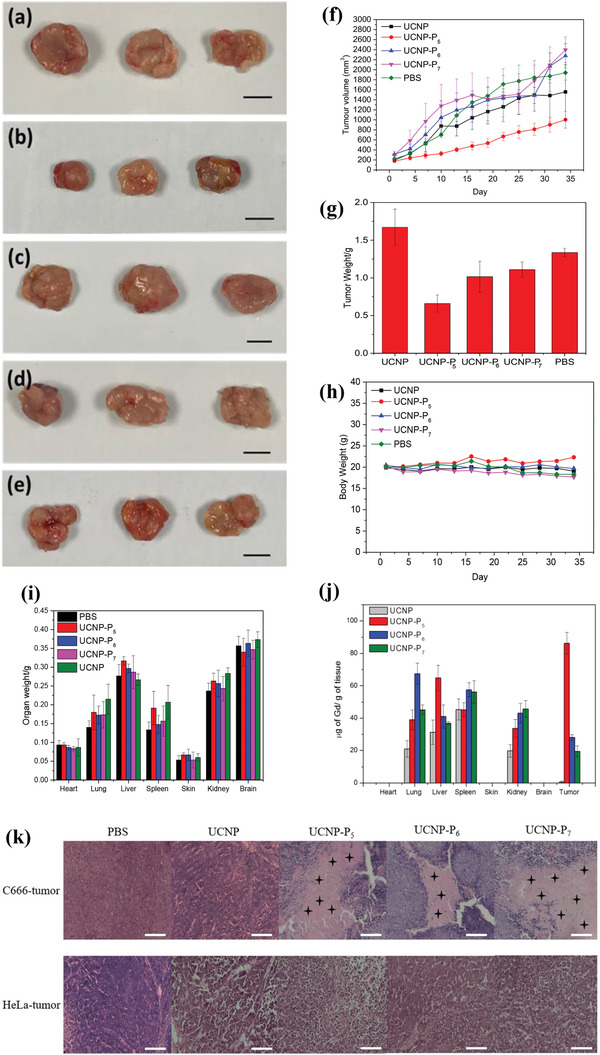
a) Digital photographs of tumor after treatment of a) PBS b) UCNP‐P_5_ c) UCNP‐P_6_ d) UCNP‐P_7_ e) UCNP. Scale bar: 10 mm; f) Tumor volume after treatment of UCNP, UCNP‐P_5_, UCNP‐P_6_, UCNP‐P_7,_ and PBS during 33 day experimental period. g) Tumor weight after treatment of UCNP, UCNP‐P_5_, UCNP‐P_6_, UCNP‐P_7_ and PBS during 33 day experimental period. h) Body weight in UCNP, UCNP‐P_5_, UCNP‐P_6_, UCNP‐P_7_ and PBS group after experimental period of 33 days i) Weights of vital organs, i.e., heart, lung, liver, spleen, skin, kidney, and brain in UCNP, UCNP‐P_5_, UCNP‐P_6_, UCNP‐P_7_ and PBS group after experimental period of 33 d; j) ICP‐MS results in different organs and tumor by detecting Gd ions after treatment of UCNP and UCNP‐P*_n_* (*n* = 5, 6, and 7). k) H&E‐stained sections of C666 and HeLa derived tumors after different treatments including PBS, UCNP, UCNP‐P_5_, UCNP‐P_6,_ and UCNP‐P_7_. The black star‐like labels indicate cell necrosis in the tumors. Scale bar = 100 µm.

To determine the antitumor efficacy of the new nanoprobes in vivo, their therapeutic effects were investigated in C666 and HeLa derived xenografts in BALB/c nude mice. The mice were randomly divided into five groups: 1) control (PBS), 2) UCNP, 3) UCNP‐P_5_, 4) UCNP‐P_6_, 5) UCNP‐P_7_. In order to recapitulate the clinical setting of drug delivery, intravenous injection (via a tail vein) was used to deliver these agents to the immunocompromised animal. The preliminary results have shown that 12.5 mg kg^−1^ UCNP‐P_5_ could significantly reduce the average tumor size of the C666‐derived tumor in nude mice (**Figure** **7**a–g). In contrast, the same amount of UCNP‐P_6,_ or UCNP‐P_7_ had no obvious effect on the tumor size when compared with the solvent and the UCNP‐alone (without any EBNA‐LMP1 binding peptide) controls, slight reduction of body weight was observed in these animals (Figure 7h). With the UCNP‐P_5_ treatment, the average body weight and various organ weights slightly increased during the experimental period (Figure 7h,i), these data can be interpreted as an indicator for the drug efficacy and safety. On the other hand, the in vivo biodistribution detected by ICP‐MS has shown that UCNP‐P_5_ was taken up by the tumor approximately threefold higher than UCNP‐P_6,_ and UCNP‐P_7_, and UCNP‐alone was nearly undetectable in the tumor (Figures 7j). This can explain why UCNP‐P_5_ is the most effective UCNP‐associated compound when delivered through the circulation, and also indicating that the conjugated peptide contributes to its tumor‐specificity. In addition, partial accumulation of nanoprobes in the reticuloendothelial system (RES), such as liver, spleen and lung, where UCNP‐P_5_ had been taken up, but their uptake values were less than the tumors after injection which inferred that excretion occurred. None of our nanoprobes had any significant effect on the tumor growth of HeLa (Figure S45, Supporting Information), suggesting that UCNP‐P_5_ is a specific agent towards EBV‐associated tumors via targeting EBNA1 and LMP1, with desired safety and therapeutic effectiveness.

In vivo upconversion luminescence imaging was performed using UCNP‐P_5_ under 980 nm excitation (laser power: 3 W). C666‐tumor‐bearing and HeLa‐tumor‐bearing nude mice were injected with UCNP‐P_5_ through the tail vein. Upconversion luminescence images of the mice were collected at different time intervals of 3, 6, 12, 24, 36 h after the injection, and green emission signals from tumors were recorded (Figure S46, Supporting Information). In nude mice with a HeLa derived xenograft, the emission signal of UCNP‐P_5_ from the tumor site was only observed at 3 h after the injection, the signal declined dramatically and was absent afterwards. In sharp contrast, UCNP‐P_5_ showed a lasting upconversion luminescence signal from the tumor site, which decayed with time but persisted even 24 h after the injection in C666‐tumor‐bearing nude mice. This result manifested that UCNP‐P_5_ possessed specific targeting capability in EBV‐positive tumor, which induced the accumulation and retention enhancement of nanoplatforms in the C666‐tumor.

Moreover, hematoxylin and eosin (H&E) staining of C666 and HeLa tumor sections showed that only C666 cancer cells and vasculature were noticeably damaged with UCNP‐P*_n_* (*n* = 5, 6, and 7) treatment (Figure 7k), which implied that cell necrosis occurred inside the tumor. Meanwhile, the PBS and UCNP groups exhibited negligible damage in C666 derived xenografts. In sharp contrast, tightly packed cancer cells were observed among all experimental groups in the HeLa derived tumors. In addition, the H&E histological analysis of major organs in C666‐tumor‐bearing and HeLa‐tumor‐bearing nude mice indicated that UCNP‐P*_n_* (*n* = 5, 6, and 7) did not cause any side effects and pathological abnormalities (Figures S47 and S48, Supporting Information), indicating the satisfactory safety and biocompatibility of the novel nanoplatforms.

## 3 Conclusions

We have designed and synthesized dual‐EBV‐oncoproteins‐targeting and pH‐responsive luminescent nanoprobes with responsive upconversion emission for precision targeting, monitoring, and inhibition of EBV‐associated cancer. A pH‐responsive linker prepared by Schiff base reaction was introduced to link the UCNP and dual‐EBNA1/LMP1‐targeting peptide. Such a rational design not only empowered the nanoprobes to be released once entering the tumor cell after attachment by the LMP1‐specific motif, but could also reduce the undesired side effects on normal tissues. Furthermore, our nanoprobe UCNP‐P_5_ was also able to display specific and sensitive emission enhancement responses toward EBV‐positive cell lines. Notably, selective cytotoxicity toward EBV‐infected cancer cells was achieved by the EBNA1‐specific motif, and was further enhanced by targeting LMP1. More importantly, the therapeutic efficacy of UCNP‐P_5_ was clearly demonstrated by the in vivo inhibition of EBV‐positive tumors and the enhanced specific uptake. This study has opened a new avenue for precision biomedical application using a dual‐functional peptide. In addition, the nanoplatform can function as an imaging agent due to its responsive photophysical properties. Results of this study have demonstrated the successful use of EBV proteins as drug targets, and we envisage our dual‐targeting peptide‐guided approach could be conveniently translated and applied to other cancers.

## 4 Experimental Section

### Chemicals and Reagents

Gadolinium(III) acetate hydrate, ytterbium (III) acetate hydrate and erbium(III) acetate hydrate (99.9% trace metals basis), human serum albumin (HSA), avidin, bovine serum albumin (BSA), nucleophosmin (NPM), ammonium fluoride (NH_4_F), cyclohexane, dimethyl sulfoxide (DMSO), ethanol, hydrochloric acid (HCl), methanol, oleic acid (OA), 1‐octadecene (ODE), sodium hydroxide (NaOH), and triethylamine (TEA) and were purchased from Sigma–Aldrich and used without purification. SH‐PEG_2k_‐CHO was purchased from Shanghai Yuanyang Biotech Co., Ltd. Metal salt solutions were prepared in deionized water; NH_4_F and NaOH solutions were prepared in methanol.

### Synthesis of NaGdF_4_: Yb^3+^, Er^3+^ Core Upconversion Nanoparticles

Core NaGdF_4_:Yb^3+^, Er^3+^ was fabricated through the coprecipitation method which followed the reported synthetic steps.^[^ ^47^ ^]^ 4 mL OA, 6 mL ODE and gadolinium(III) acetate hydrate (0.312 × 10^−3^ m), ytterbium (III) acetate hydrate (0.08 × 10^−3^ m) and erbium(III) acetate hydrate (0.008 × 10^−3^ m) were mixed and stirred at 150 °C for 40 min, followed by the addition of methanolic solutions of 1.6 × 10^−3^ m NH_4_F and 1 × 10^−3^ m NaOH. The mixture was then stirred at 50 °C for 30 min and subsequently put under vacuum at 100 °C for 10 min. Then, the reaction flask put under a nitrogen atmosphere and heated at 290 °C for 90 min. The reaction mixture was then cooled to room temperature, washed with cyclohexane and ethanol (twice) and the product was collected via centrifugation.

### Synthesis of NaGdF_4_: Yb3+, Er3+@NaGdF4 Core–Shell Upconversion Nanoparticles

First, 2 mL of gadolinium(III) acetate hydrate (0.2 m), 4 mL OA and 6 mL ODE were mixed in a 50 mL flask and then maintained at 150 °C for 40 min to yield the shell precursors. The core nanoparticles, 5 mL NaOH (1 × 10^−3^ m) and NH_4_F (1.6 × 10^−3^ m) were injected after cooling down to 50 °C and the reaction mixture was stirred at 50 °C for 30 min, then vacuuming at 100 °C for 10 min. Upon restoration of a nitrogen atmosphere, the reaction mixture was heated at 280 °C for 1 h and then cooled to room temperature. Ethanol was added to precipitate the resultant nanoparticles and they were collected and washed with ethanol through centrifugation at 6000 rpm for 3 min.

### Synthesis of Hydrophilic Aldehyde‐Functionalized Core–Shell Upconversion Nanoparticles

The UCNP‐CHO with good biocompatibility were synthesized by strong thiol‐metal interactions between the Gd^3+^ ion and thiol group. 0.1 m HCl was added to the nanoparticles and were sonicated at 50 °C for 1 h to get rid of the oleic ligand on surface. The ligand‐free nanoparticles were gathered after centrifugation at 14 000 rpm for 30 min and then redispersed in deionized water. 10 mL of SH‐PEG_2k_‐CHO (200 mg) was added to the nanoparticles and the mixture was stirred slowly at room temperature for 24 h. Centrifugation at 14 000 rpm for 30 min was performed again to remove excess SH‐PEG_2k_‐CHO and the UCNP‐CHO was obtained.

### Coating of Dual‐EBNA1/LMP1‐Targeting Specific Peptide on the UCNP

The UCNP‐P*_n_* (*n* = 5, 6, and 7) were synthesized through a Schiff base reaction. Briefly, UCNP‐CHO (100 mg), dual‐targeting protein specific peptide P*_n_* (*n* = 5, 6, and 7) (80 mg) and 21 µL of triethylamine (TEA) were codissolved in DMSO (2 mL). The mixed solution was maintained for 24 h at 40 °C under magnetic stirring at 300 rpm. The mixture was rinsed with methanol to remove extra peptide and UCNP‐P*_n_* (*n* = 5, 6, and 7) was obtained after freeze drying.

### Characterization

The as‐prepared UCNP and UCNP‐P*_n_* (*n* = 5, 6, and 7) were characterized with transmission electron microscope (TEM), X‐ray diffraction (XRD), Fourier transform infrared spectra (FTIR), dynamic light scattering (DLS), and zeta‐potential measurements using the same equipment with the previous work.^[^ ^33^ ^]^ Visible emission spectra of UCNP and UCNP‐P*_n_* (*n* = 5, 6, and 7) were measured using a SpectroFluorometer System (Horiba/Fluoromax‐4) equipped with a 980 nm laser as the excitation source. UV–vis absorbance measurements were recorded by Agilent Technologies Cary 8454 UV–Vis machine.

### Cell Culture

HK1‐LMP1 was generated by stably expressing the EBV oncoprotein, latent membrane protein1 (LMP1) variant (B95.8) into the parental HK1 cell line by transfection. This cell line was established and cultured as previously described.^[^ ^48^ ^]^ Other cell lines were obtained from the same affiliations in the previous work.^[^ ^33^ ^]^

LMP1‐positive HK1‐LMP1 cells and LMP1‐negative HK1 cells were grown in Dulbecco's modified Eagle medium (DMEM); LMP1‐positive LCL3, Raji cells were maintained in RPMI 1640 medium. The media used in cell culture of NPC43, HeLa, C666 and MRC‐5 cells were consistent with the one previously reported.^[^ ^33^ ^]^ 10% fetal bovine serum (FBS), 1% penicillin, and streptomycin were all used to supplement the media used.

### In Vitro Confocal Imaging and Nuclear Localization Imaging

The cells were cultured in 35 mm cell culture dishes for 24 h prior to incubation in dark with 30 µg mL^−1^ of UCNP and UCNP‐P*_n_* (*n* = 5, 6, and 7) for different time intervals (1, 3, 6, 12, and 24 h). The cells were then costained with 0.05 × 10^−6^ m nuclear dye DRAQ5 for 0.5 h for confocal imaging with a Leica SP8 confocal microscope equipped with a coherent femtosecond laser (690–1050 nm), argon laser (432, 457, and 488 nm), He–Ne laser (632 nm), UV lamp. A stage‐top cell culture chamber was also used to maintain the atmospheric conditions of 2–7% CO_2_ and a temperature of 37 °C.

### Western Blotting

EBV‐positive C666, LCL3 and EBV‐negative HK1 cell pellets were treated with phosphatase and protease inhibitors at 0–4 °C for 30 min. The total protein concentrations were calculated by protein absorbance. The protein was resolved by sodium dodecyl sulfate‐polyacrylamide gel electrophoresis (SDS‐PAGE) and transferred to cellulose filter membranes. The membranes were blocked with 3% BSA in tris‐buffered saline in 0.1% Tween 20 (TBST) for 1 h with gentle shaking and subsequently incubated with primary antibodies (1:1000) in 3% BSA in TBST at 4 °C for overnight. After rinsing with TBST buffer three times, the blots were then incubated with corresponding secondary antirabbit antibody (1:6000) for 1 h. After that, the blots were rinsed again with 3% BSA in TBST for 1 h shaking at room temperature. Lastly, the density of bands was determined by image analysis system after washing with TBST buffer three times.

### Cellular Uptake of Nanoprobes by ICP‐MS

To examine the intracellular concentration of nanoprobes in different cell lines, namely C666, HK1, HK1‐LMP1, LCL3 cells, 1 × 10^5^ cells were plated in each well in six‐well plates and incubated with UCNP, UCNP‐P*_n_* (*n* = 5, 6, and 7) and the old nanoprobe UCNP‐P_4_ at 50 µg mL^−1^ for different time intervals of 1, 3, 6, 12, and 24 h. After coincubation, the cell culture medium containing the nanoprobes was removed, and the exposed cells were further rinsed with 1 mL PBS three times. After that, the cells were trypsinized and redispersed in 1 mL cell medium. The cells were collected by centrifugation at 1500 rpm, and the cell pellet was digested in 100 µL concentrated HNO_3_ (69%) at room temperature for 3 h. Then, each cell pellet in centrifuge tube was filled up to 10 mL with 1% HNO_3_. The cellular uptake of all the nanoprobes was recorded via an Agilent 7500 series ICP‐MS instrument. All ICP experiments were performed three times, and the values of Gd concentration (ppm) obtained were calculated and averaged.

### In Vitro Cytotoxic Assays

The cytotoxicity of our nanoprobes was determined by MTT reduction assay. The cells (3 × 10^3^ per well) were seeded onto 96‐well plates and incubated overnight, followed by treatment with P*_n_* (*n* = 5, 6, and 7), UCNP, UCNP‐P*_n_* (*n* = 5, 6, and 7) and UCNP‐P_4_ individually at 37 °C, 5% CO_2_ for 24 h in dark. The cell monolayers were rinsed with PBS and 50 µL of MTT, 3‐(4,5‐dimethylthiazol‐2‐yl)‐2,5‐diphenyltetrazolium bromide solution (0.5 mg mL^−1^) was added per well and the cells were further incubated for 3 h at 37 °C. The solution was subsequently discarded, and the formazan crystals were dissolved in 100 µL of DMSO per well under shaking. The absorptions of the formazan crystal solutions at 540 and 690 nm were measured using a dual‐wavelength Labsystem Multiskan microplate reader (Merck Eurolab).

### Dimerization Inhibition Assay

90 µg of the EBNA1 (DNA‐binding domain fragment of a.a.468‐607) was first incubated with UCNP and UCNP‐P*_n_* (*n* = 5, 6, and 7) (conc.: 0.3 µg µL^−1^) at 4 °C overnight. Then, 2 × 10^−3^ m disuccinimidyl suberate (DSS) was added to allow the samples to undergo crosslinking reaction at room temperature for 30 min. The samples were run and separated on an SDS‐denaturing gel and proteins were detected using Coomassie Blue staining. PBS buffer was used as control group.

### Modelling and Simulation of Peptides

The designed peptides were docked into the dimerization interface of a putative EBNA1 monomer reported in previous study (obtained from Protein database ID: 1B3T) using HADDOCK version 2.2. The parameters reported for nonstandard residue (Ahx) was used in this simulation. The system was described using ff14SB variant force field. After solvation with 10 Å explicit TIP3P water box, the system was minimized and proceed to unbiased MD simulation using GPU version of PMEMD engine in Amber 16 Software Package. All systems were heated from 100 to 300 K in 1 ns. The system was further equilibrated for 1 ns with constant pressure and temperature before proceeding to the 200 ns NPT production stage. SHAKE‐enabled setting to constrict hydrogen bonds were used for all equilibration and production stages. Langevin thermostat was used to control the temperature throughout the simulations.

### Postsimulation Analysis

The conformational clusters of EBNA1‐complex were obtained using default settings with distance defined by C_a_ atoms root mean square deviation (RMSD) by using cpptraj. The most abundant cluster for all complexes were used for interaction analysis. The binding free energies between protein and peptides were calculated using molecular mechanics Poisson‐Boltzmann surface area (MMPBSA) method. The whole production trajectory for all systems were used. The salt concentration was set to 0.1 m in GB calculation.

### In Vivo Suppression Assays

EBV‐positive C666 and EBV‐negative HeLa cells were suspended in 200 µL of serum‐free RMPI 1640 and DMEM respectively. Female BALB/c nude mice (6–8 weeks) which obtained from HKU were then injected with the cells in the right flank. Intravenous injections were performed when the average tumor volumes reached ≈200 mm^3^. 0.25 mg per tumor dose of UCNP and UCNP‐P*_n_* (*n* = 5, 6, and 7) in 100 µL PBS buffer were injected through tail vein of mice using a 24‐gauge syringe. Mice injected with the same volume of PBS buffer served as control. The body weight and tumor volumes of the mice were measured three times per week; in which the latter is calculated by (*L* × *W* ^2^)/2, where *L* and *W* are the longer and shorter tumor dimensions respectively. Intravenous injection at the tail vein were performed twice a week and the mice were sacrificed after 33 day experimental period; the tumors were extracted and weighed. The treatment groups are unknown to the investigators for the experimental and data analysis processes. The animal experiments conducted were approved by the Department of Health of the HKSAR Government. All animal procedures are within the Guidelines for Care and Use of Laboratory Animals of HKBU and approved by the Animal Ethics Committee of HKBU.

### In Vivo Upconversion Luminescence Imaging

C666 and HeLa cells (10^6^ cells) were inoculated subcutaneously in BALB/c female nude mice (6 weeks). When the tumors reached 0.2−0.3 cm in diameter, the C666‐tumor‐bearing and HeLa‐tumor‐bearing mice were anesthetized and injected intravenously with UCNP‐P_5_ (150 µL, 12.5 mg kg^−1^ per mouse). The in vivo upconversion luminescence images were recorded at different time points (3, 6, 12, 24, 36 h).

### Hematoxylin and Eosin (H&E) Staining

In general, the transplanted C666 and HeLa derived tumor tissues were collected and fixed with formalin, followed by embedding in paraffin. The histologic sections were then stained with hematoxylin and eosin. Other major organ tissues (heart, liver, spleen, lung, kidney, brain) were conducted in the same procedures.

### Analytical HPLC

Analytical HPLC was performed on an Agilent 1100 series HPLC system (Agilent Technologies, Stockport, UK) equipped with a diode‐array detection (DAD) detector and Agilent C18 column (250 mm x 4.6 mm) for corresponding peptides (P_5_, P_6_, P_7_) at the following gradient:

**Table:** 

Time	A % (H_2_O + 0.1% TFA)	B % (MeCN + 0.1% TFA)	Flow
0	80	20	0.5
40	20	80	0.5
41	0	100	0.5
50	0	100	0.5

### Statistical Analyses

All experiments were conducted in triplicate. All experimental data were based on independent experiments and presented as the mean ± standard deviation (SD) (*n* = 3). Student's *t*‐test was employed for statistical analysis by using Origin 2016. Difference with *P* < 0.05 (*) or *P* < 0.01(**) was considered statistically significant.

## Conflict of Interest

The authors declare no conflict of interest.

## Supporting information

Supporting Information
