# Supplementary material for: Dual‐Targeting Peptide‐Guided Approach for Precision Delivery and Cancer Monitoring by Using a Safe Upconversion Nanoplatform
Source: Adv Sci (Weinh). 2021 Jan 6;8(5):2002919. doi: 10.1002/advs.202002919 (PMC7927616; doi:10.1002/advs.202002919)
Supplement: Supplementary file 1 — Supporting Information [file ADVS-8-2002919-s001.pdf]

WILEY-VCH

© 2020 Wiley-VCH GmbH

ADVANCED SCIENCE
Open Access

Supporting Information

for *Adv. Sci.,* DOI: 10.1002/advs.202002919

Dual-targeting Peptide-guided Approach for Precision Delivery and Cancer Monitoring by using a Safe Upconversion Nanoplatform

Shuai Zha, Ho-Fai Chau, Wai Yin Chau, Lai Sheung Chan, Jun Lin, Kwok Wai Lo, William Chi-shing Cho, Yim Ling Yip, Sai Wah Tsao, Paul J. Farrell, Liang Feng, Jin Ming Di, Ga-Lai Law,* Hong Lok Lung,* and Ka-Leung Wong**

© 2020 Wiley-VCH GmbH

Supporting Information

Dual-targeting peptide-guided approach for precision delivery and cancer monitoring by using a safe upconversion nanoplatform

Shuai Zha, Ho-Fai Chau, Wai Yin Chau, Lai Sheung Chan, Jun Lin, Kwok Wai Lo, William Chi-shing Cho, Yim Ling Yip, Sai Wah Tsao, Paul J. Farrell, Liang Feng, Jin Ming Di, Ga-Lai Law,* Hong Lok Lung,* and Ka-Leung Wong**

Shuai Zha, Ho-Fai Chau, Dr. Lai Sheung Chan, Dr. Hong Lok Lung, Prof. Ka-Leung Wong
Department of Chemistry, Hong Kong Baptist University, 224 Waterloo Road, Kowloon, Hong Kong S.A.R., P. R. China
E-mail: hllung2@hkbu.edu.hk (H. L. L.)
E-mail: klwong@hkbu.edu.hk (K.-L.W.)

Wai Yin Chau
Department of Biology, Hong Kong Baptist University, 224 Waterloo Road, Kowloon, Hong Kong S.A.R., P. R. China

Prof. Jun Lin
State Key Laboratory of Rare Earth Resource Utilization, Changchun Institute of Applied Chemistry, Chinese Academy of Sciences, Changchun, P. R. China

Prof. Kwok Wai Lo
Department of Anatomical & Cellular Pathology and State Key Laboratory of Translational Oncology, The Chinese University of Hong Kong, Hong Kong S.A.R., P. R. China

Dr. William Chi-shing Cho
Department of Clinical Oncology, Queen Elizabeth Hospital, Kowloon, Hong Kong S.A.R., P. R. China

Dr. Yim Ling Yip, Prof. Sai Wah Tsao
School of Biomedical Sciences, The University of Hong Kong, Hong Kong S.A.R., P. R. China

Prof. Paul J. Farrell
Section of Virology, Imperial College Faculty of Medicine, Norfolk Place, London, United Kingdom

Dr. Jin Ming Di
Department of Urology, The Third Affiliated Hospital of Sun Yat-sen University, 600# Tianhe Road, 510630 Guangzhou, P. R. China
E-mail: 746379204@qq.com (J. M. D.)

Liang Feng, Dr. Ga-Lai Law

Department of Applied Biology and Chemical Technology, The Hong Kong Polytechnic University, Hung Hom, Hong Kong S.A.R., P. R. China
E-mail: ga-lai.law@polyu.edu.hk (G.-L. L.)

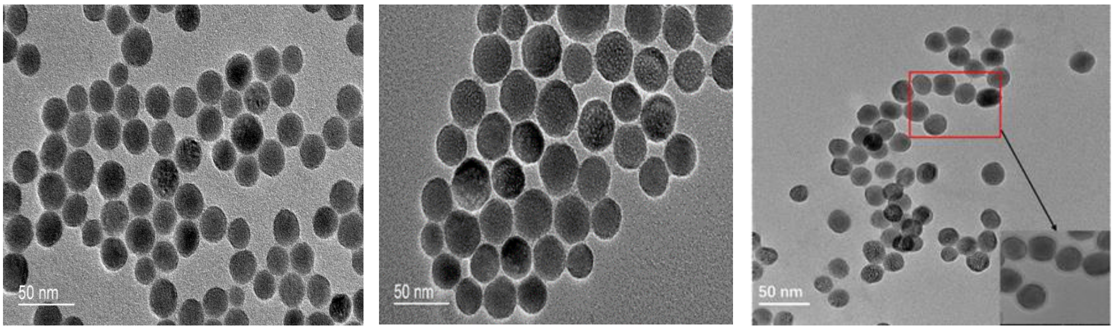

Figure S1. TEM images of (a) core upconversion nanoparticles $NaGdF_4$: Yb^{3+}, Er^{3+}, (b) $NaGdF_4$: Yb^{3+}, Er^{3+}@$NaGdF_4$ (UCNP), (c) UCNP coated with dual-targeting protein specific peptide P_5 (UCNP-P_5) (Scale bar: 50 nm)

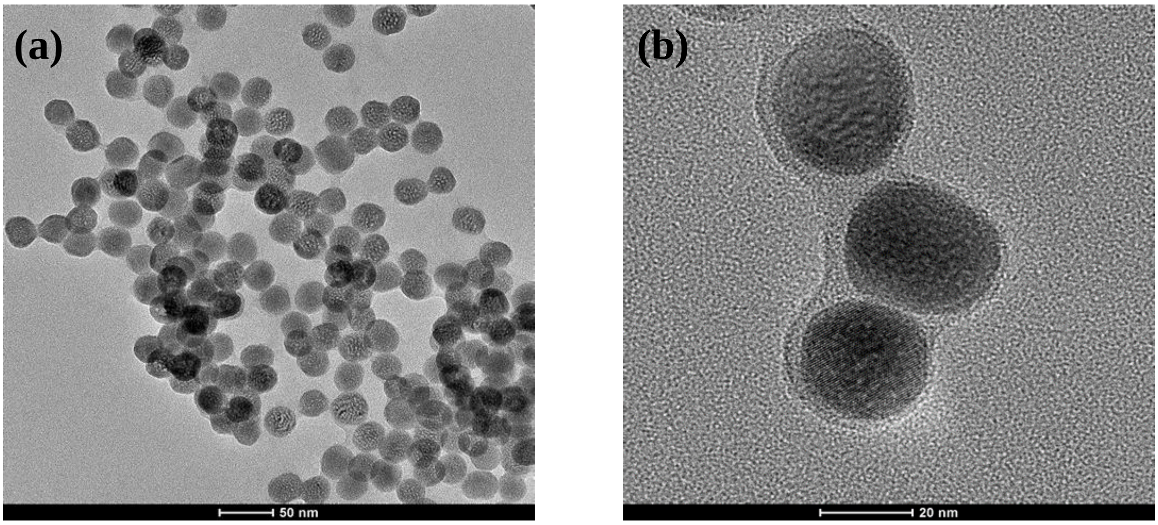

Figure S2. TEM image of UCNP coated with dual-targeting protein specific peptide P_6 (UCNP-P_6) (Scale bar: 50 nm) and (b) magnified resolution TEM image (Scale bar: 20 nm)

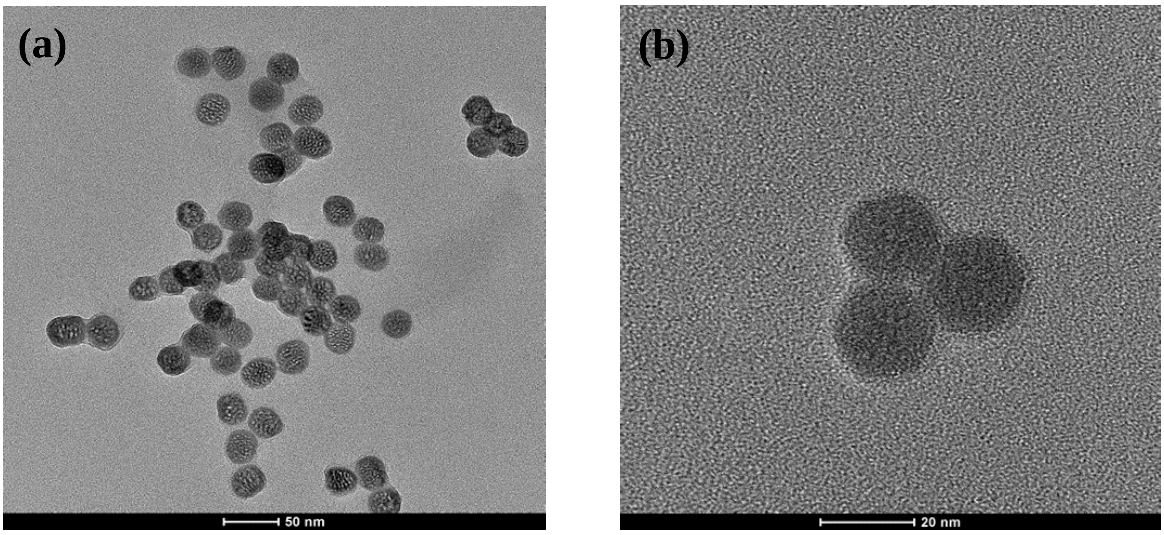

Figure S3. (a) TEM image of UCNP coated with dual-targeting protein specific peptide P_7 (UCNP-P_7) (Scale bar: 50 nm) and (b) magnified resolution TEM image (Scale bar: 20 nm)

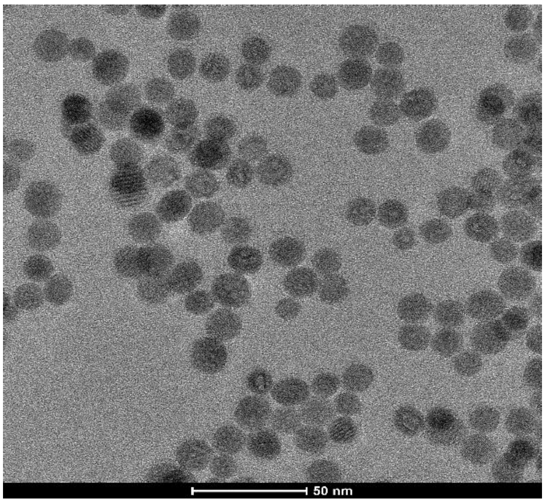

Figure S4. TEM image of UCNP coated with EBNA1 specific peptide P_4 (UCNP-P_4) (Scale bar: 50 nm)

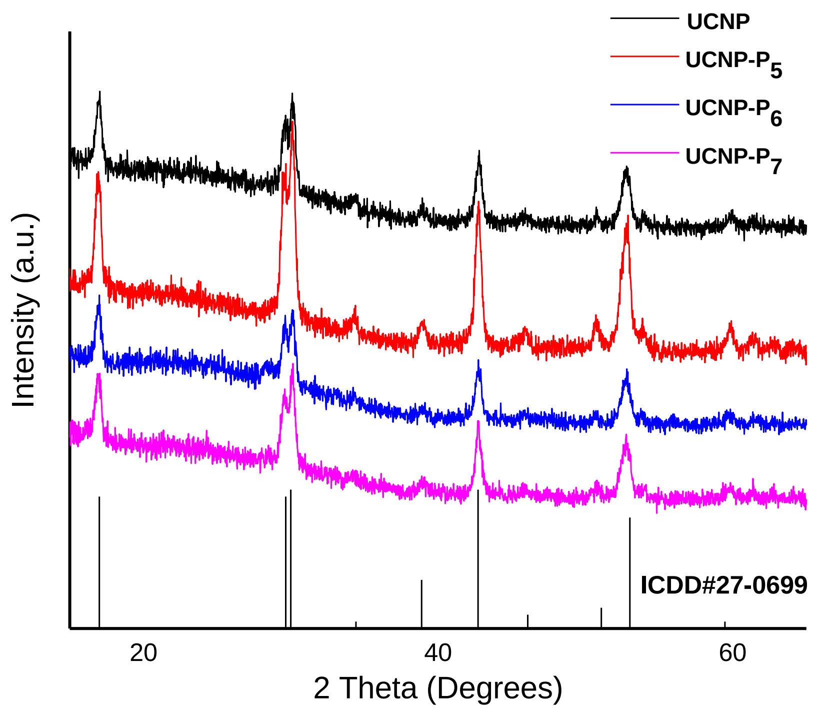

Figure S5. XRD patterns of initial nanoparticles (UCNP) and the peptide capped nanoparticles UCNP-P_n (n=5,6 and 7) indexed with a standard hexagonal-phase $NaGdF_4$ (ICDD#27-0699).

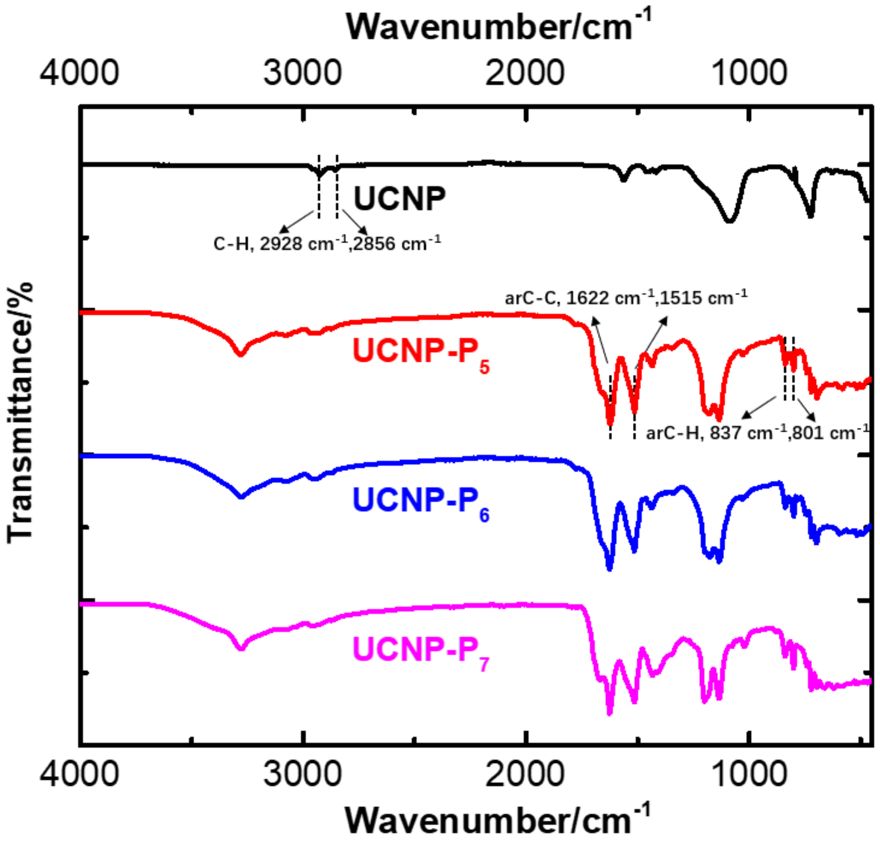

Figure S6. FTIR transmission spectrum of UCNP, dual-targeting protein specific peptides coated UCNP-P_5, UCNP-P_6 and UCNP-P_7.

(a) (b)

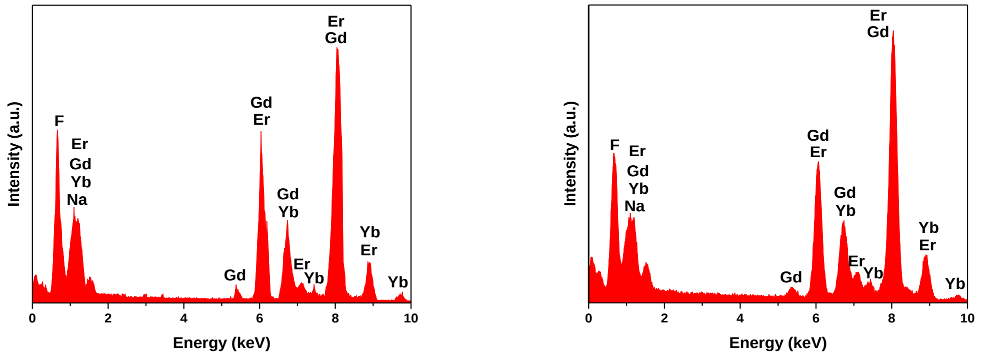

(c) (d)

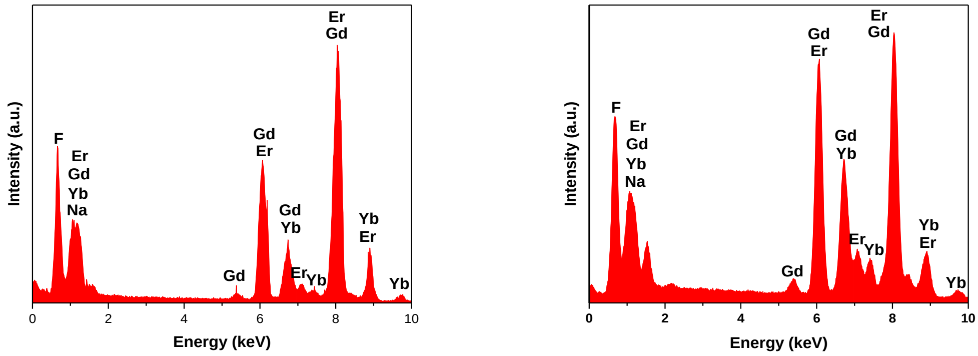

Figure S7. EDS spectra of (a) UCNP (b) UCNP-P_5 (c) UCNP-P_6 (d) UCNP-P_7.

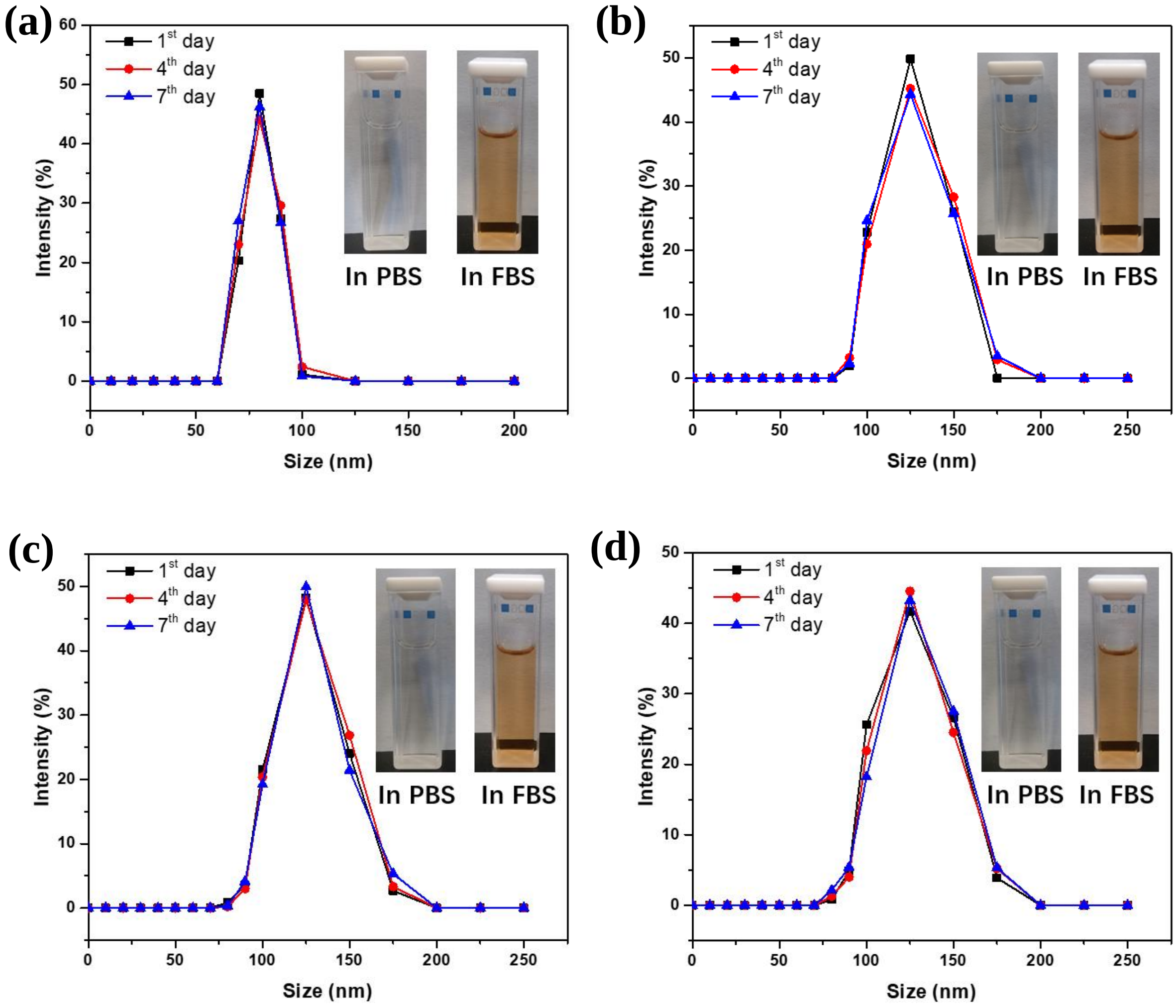

Figure S8. DLS measurements and stability studies of (a) UCNP (b) UCNP-P_5 (c) UCNP-P_6 (d) UCNP-P_7 in PBS and FBS solution. The insets in (a), (b), (c) and (d) demonstrate the corresponding digital photograph of PBS and FBS solution containing UCNP, UCNP-P_5, UCNP-P_6, and UCNP-P_7, respectively.

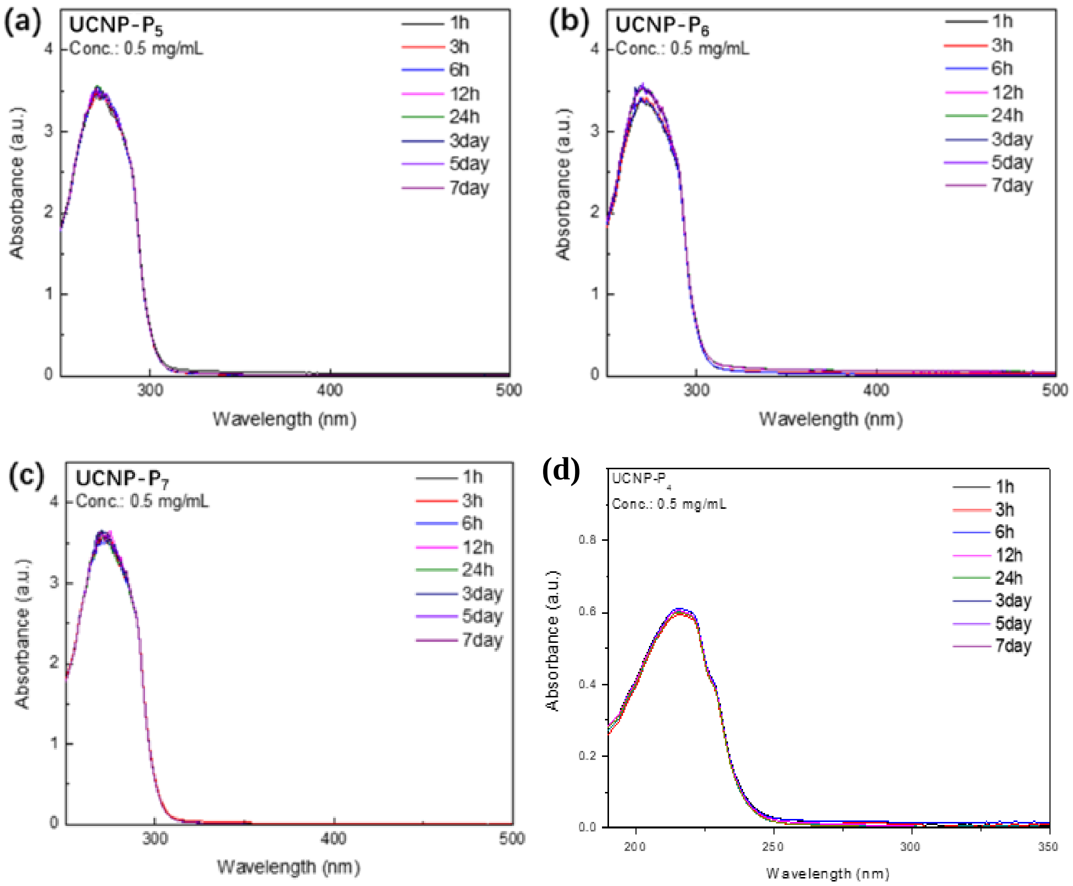

Figure S9. UV-vis absorbance spectra of (a) UCNP-P_5 (b) UCNP-P_6 (c) UCNP-P_7 (d) UCNP-P_4 under different time intervals. (Conc.: 0.5 mg/mL)

Table S1. Quantitative analysis of peptide release behaviors of nanoprobes under various time intervals. The peptide release rate for each nanoprobe at different time intervals is calculated using the following equation: $\text{release rate\%} = \frac{(I_A - I_B)}{I_A} \times 100\%$, where I_A is relative absorbance intensity at 1 h and I_B is relative absorbance intensity at other various time intervals correspondingly.

Sample names	Peptide release rates under different time intervals							
	1 h	3 h	6 h	12 h	24 h	3 day	5 day	7 day

UCNP-P_5	0%	0%	0.1%	0.5%	0.7%	0.8%	1.1%	1.9%
UCNP-P_6	0%	0%	0.3%	0.8%	1.4%	1.9%	2.9%	5.9%
UCNP-P_7	0%	0%	0.2%	0.7%	1.0%	1.6%	2.0%	2.6%
UCNP-P_4	0%	0%	0.3%	0.8%	1.3%	2.1%	3.4%	5.6%

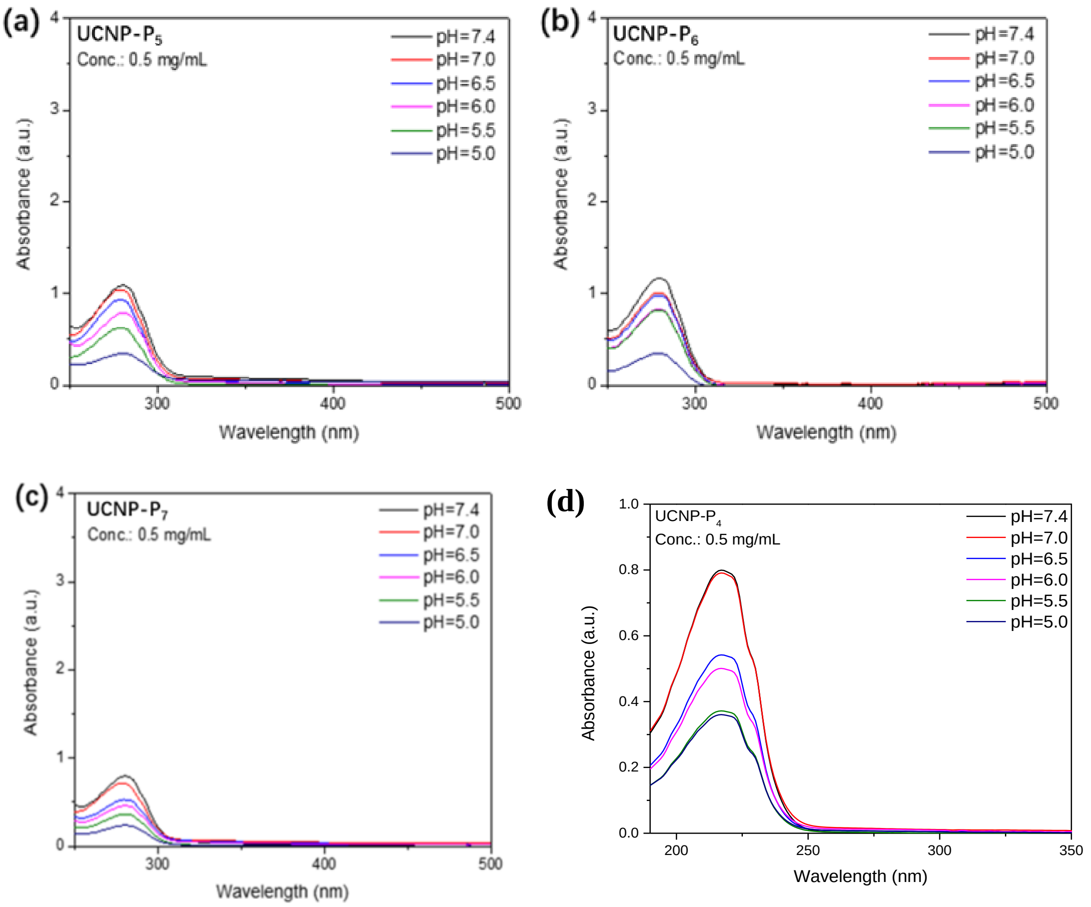

Figure S10. UV-vis absorbance spectra of (a) UCNP-P_5 (b) UCNP-P_6 (c) UCNP-P_7 (d) UCNP-P_4 under different pH buffer. (Conc.: 0.5 mg/mL). Nanoprobes were incubated in various pH buffers 24 h before measurement.

Table S2. Quantitative analysis of peptide release behaviors of nanoprobes under various pH buffers. The peptide release rate for each nanoprobe in various pH solutions is calculated using the following equation: $\text{Release rate\%} = \frac{(I_0 - I_1)}{I_0} \times 100\%$, where I_0 is relative absorbance intensity in pH 7.4 solution and I_1 is relative absorbance intensity in other different pH buffers correspondingly.

Sample names	Peptide release rates under various pH buffer					
	pH 7.4	pH 7.0	pH 6.5	pH 6.0	pH 5.5	pH 5.0
UCNP-P_5	0%	6.1%	20.9%	34.8%	56.1%	84.6%
UCNP-P_6	0%	4.6%	21.5%	32.2%	34.1%	83.3%
UCNP-P_7	0%	7.4%	36.1%	48.2%	58.5%	81.2%
UCNP-P_4	0%	1.1%	26.4%	36.5%	53.6%	57.8%

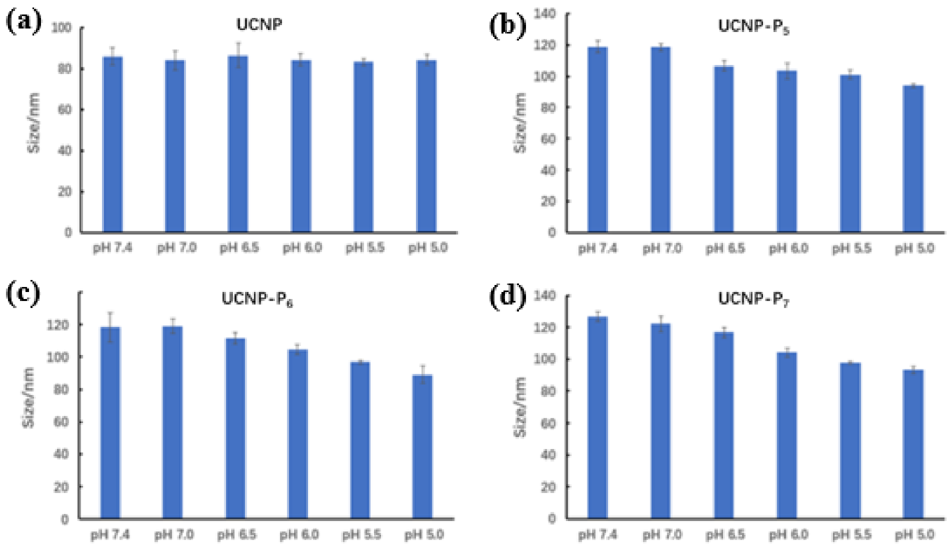

Figure S11. Size variation of nanoprobes in pH range from pH 7.4 to 5.0.

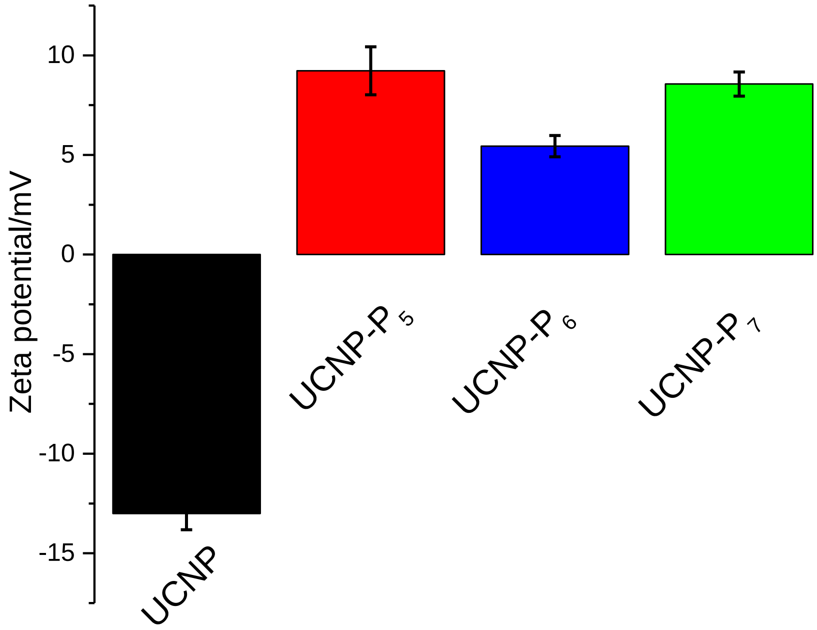

Figure S12. Zeta potential of UCNP, UCNP-P_5, UCNP-P_6 and UCNP-P_7.

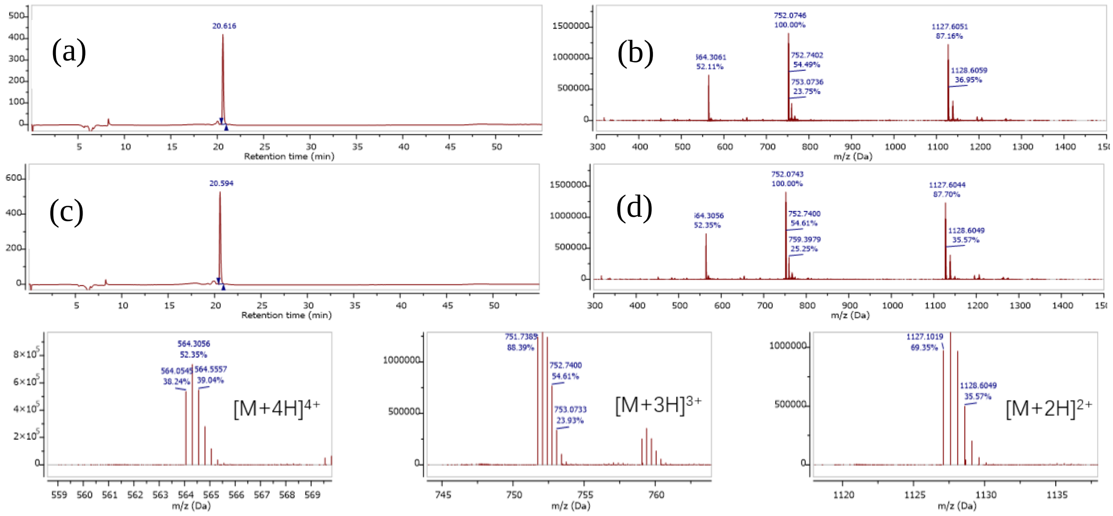

Figure S13. Analytic HPLC and ESI-MS for pre-conjugated (a-b) and post-released (c-d) P_5 sample. The retention times (T_R) are 20.616 min and 20.594 min, respectively. Molecular

formula of P_5: $C_{110}H_{157}N_{29}O_{21}S$. ESI-MS: calc. for $[M+2H]^{2+}$ 1127.0988, found 1127.1019; calc. for $[M+3H]^{3+}$ 751.7349, found 751.7385; calc. for $[M+4H]^{4+}$ 564.0530, found 564.0545.

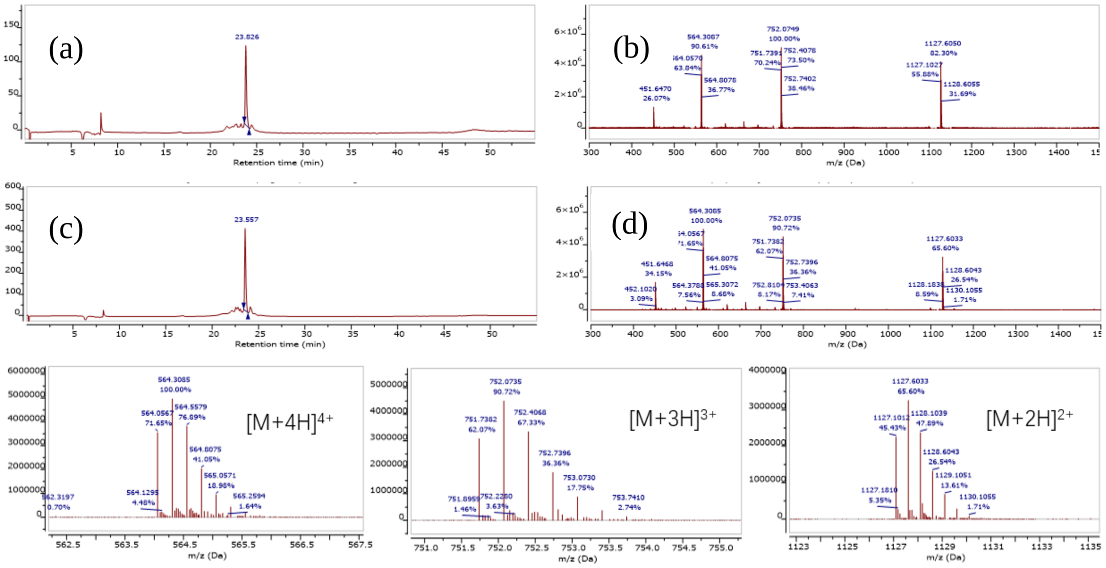

Figure S14. Analytic HPLC and ESI-MS for pre-conjugated (a-b) and post-released (c-d) P_6 sample. The retention times (T_R) are 23.826 min and 23.557 min, respectively. Molecular formula of P_6: $C_{110}H_{157}N_{29}O_{21}S$. ESI-MS: calc. for $[M+2H]^{2+}$ 1127.0988, found 1127.1012; calc. for $[M+3H]^{3+}$ 751.7349, found 751.7382; calc. for $[M+4H]^{4+}$ 564.0530, found 564.0567.

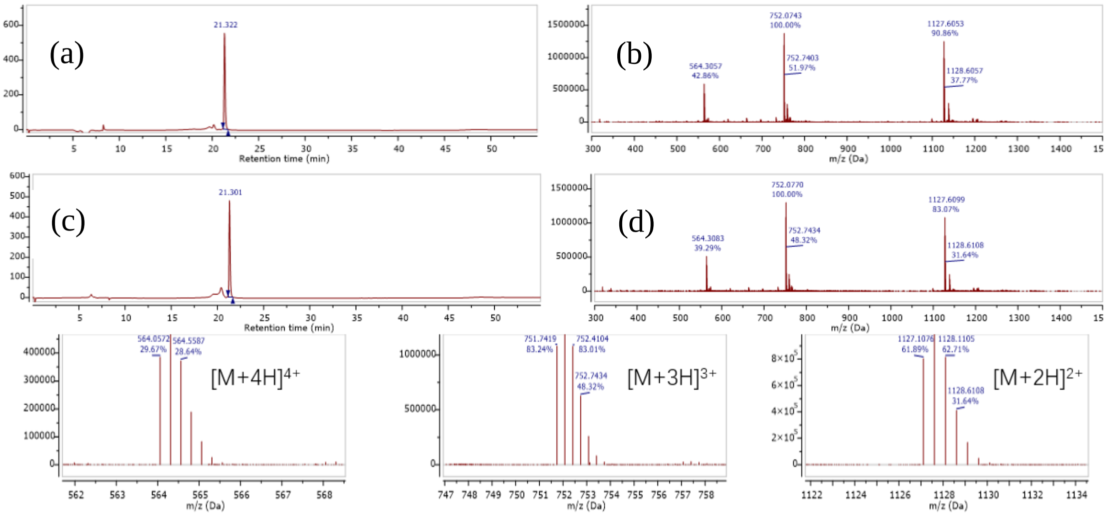

Figure S15. Analytic HPLC and ESI-MS for pre-conjugated (a-b) and post-released (c-d) P_7 sample. The retention times (T_R) are 21.322 min and 21.301 min, respectively. Molecular

formula of P_7: $C_{110}H_{157}N_{29}O_{21}S$. ESI-MS: calc. for $[M+2H]^{2+}$ 1127.0988, found 1127.1076; calc. for $[M+3H]^{3+}$ 751.7349, found 751.7419; calc. for $[M+4H]^{4+}$ 564.0530, found 564.0572.

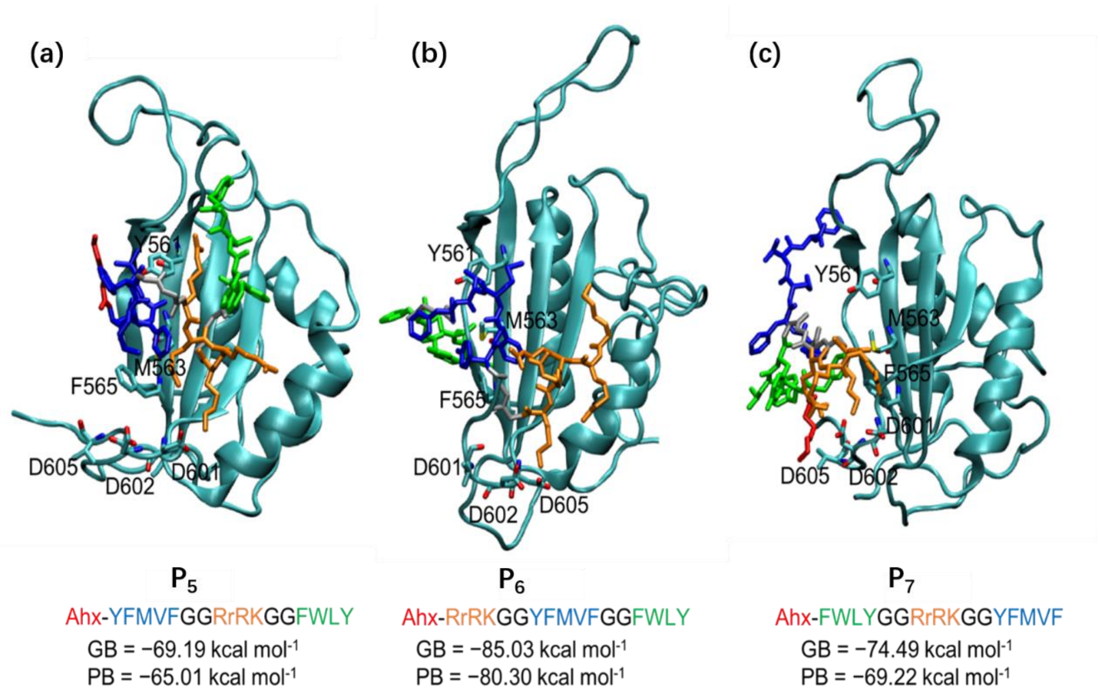

Figure S16. The representative conformations of (A) P_5, (B) P_6 and (C) P_7 with EBNA1 in the MD simulation. All binding energies calculated generalized Born (GB) and Poisson–Boltzmann (PB) values were shown for each designed peptide.

Ligand RMSD

EBNA1 RMSD
without loop

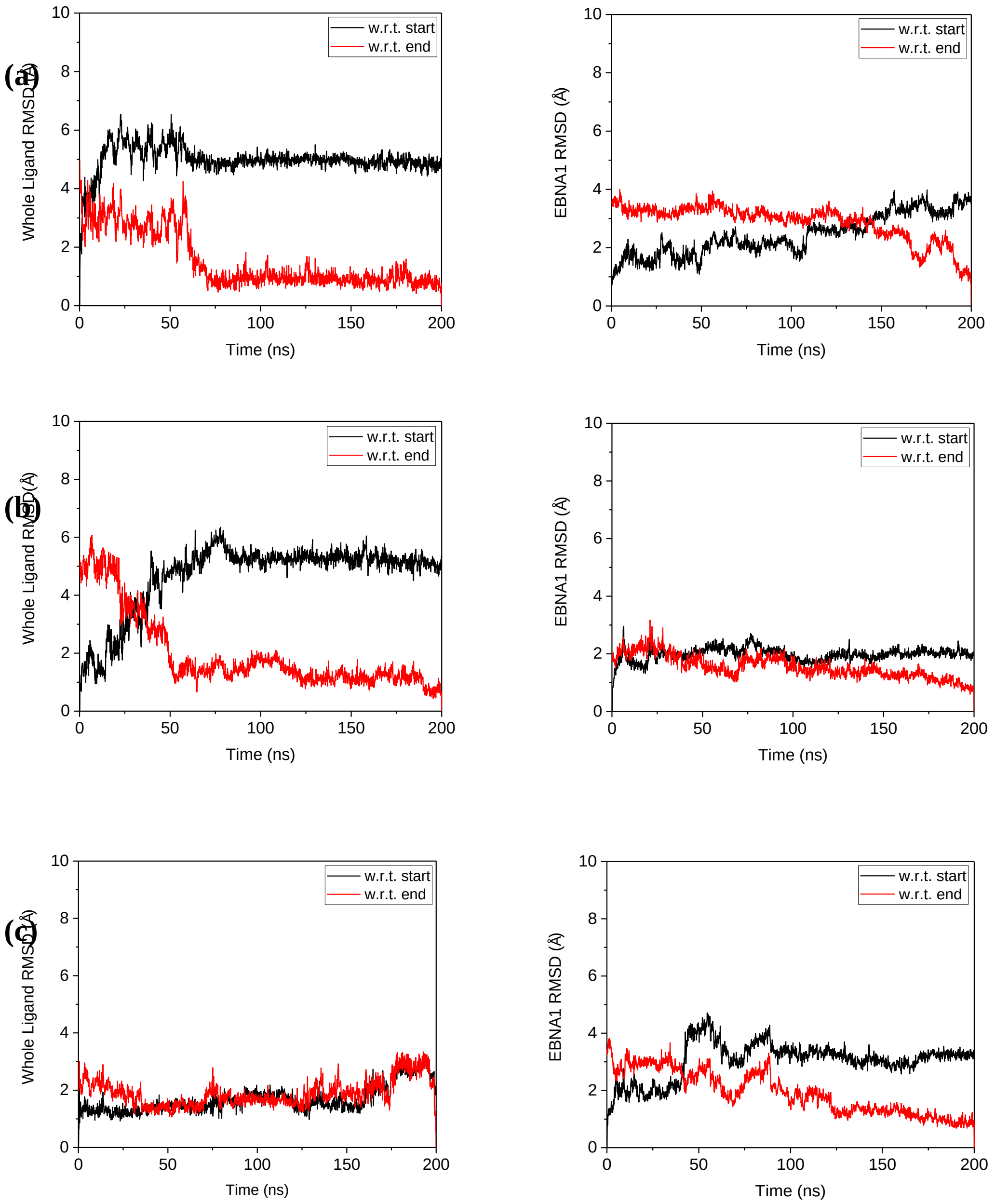

Figure S17. The RMSD of protein EBNA1 and designed peptides. The RMSD values of peptides (a) P_5, (b) P_6 and (c) P_7 with EBNA1 in the MD simulation. Black and red line represent the start and end of conformation respectively.

(a)

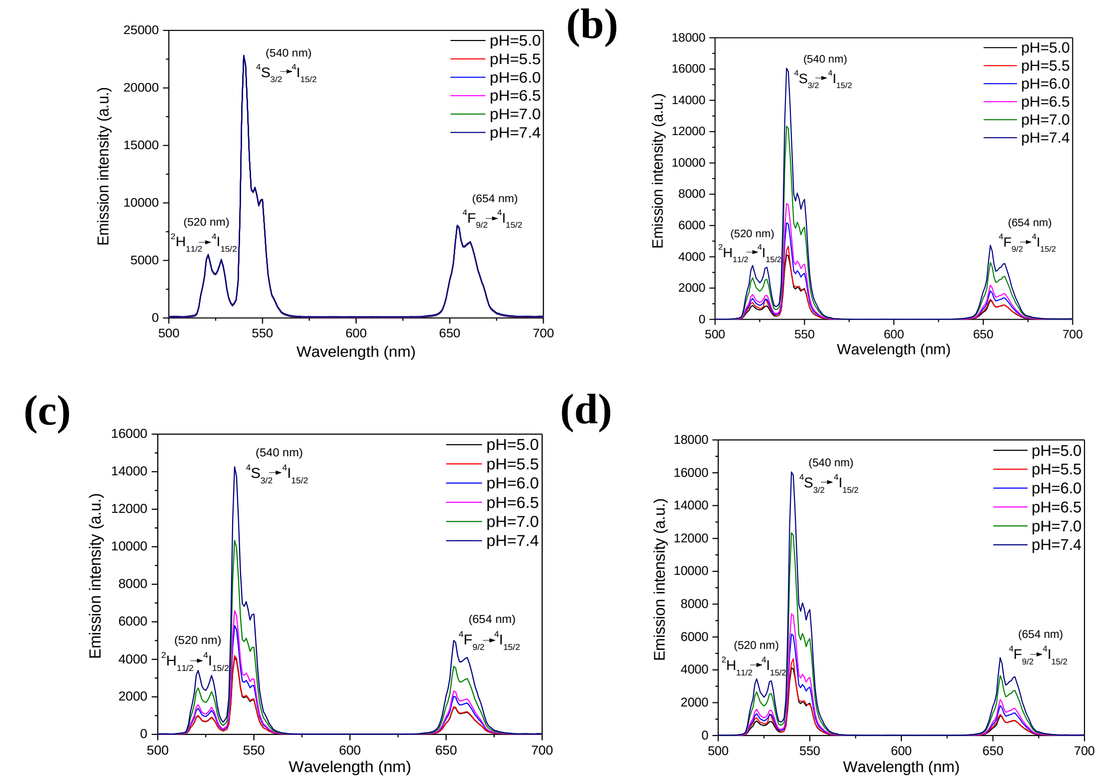

Figure S18. pH-responsive upconversion visible emission spectrum of (a) UCNP (b) UCNP-P_5 (c) UCNP-P_7. (d) UCNP-P_4.

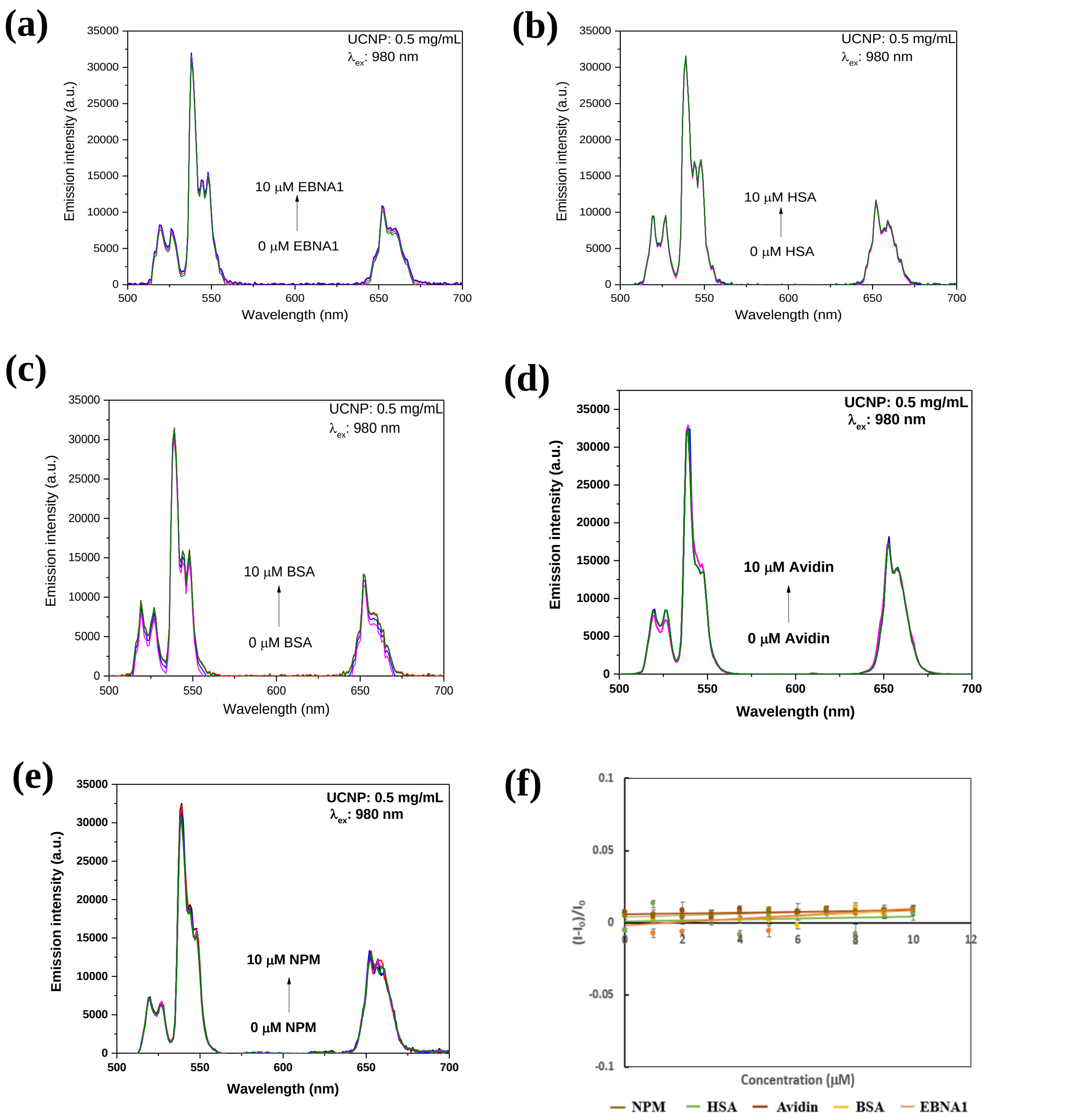

Figure S19. Luminescence titration of UCNP (conc.: 0.5 mg/mL; excitation at 980 nm) towards a) EBNA1, b) HSA, c) BSA, d) Avidin, e) NPM and f) change in emission intensity of UCNP on addition of NPM, HSA, Avidin, BSA and EBNA1.

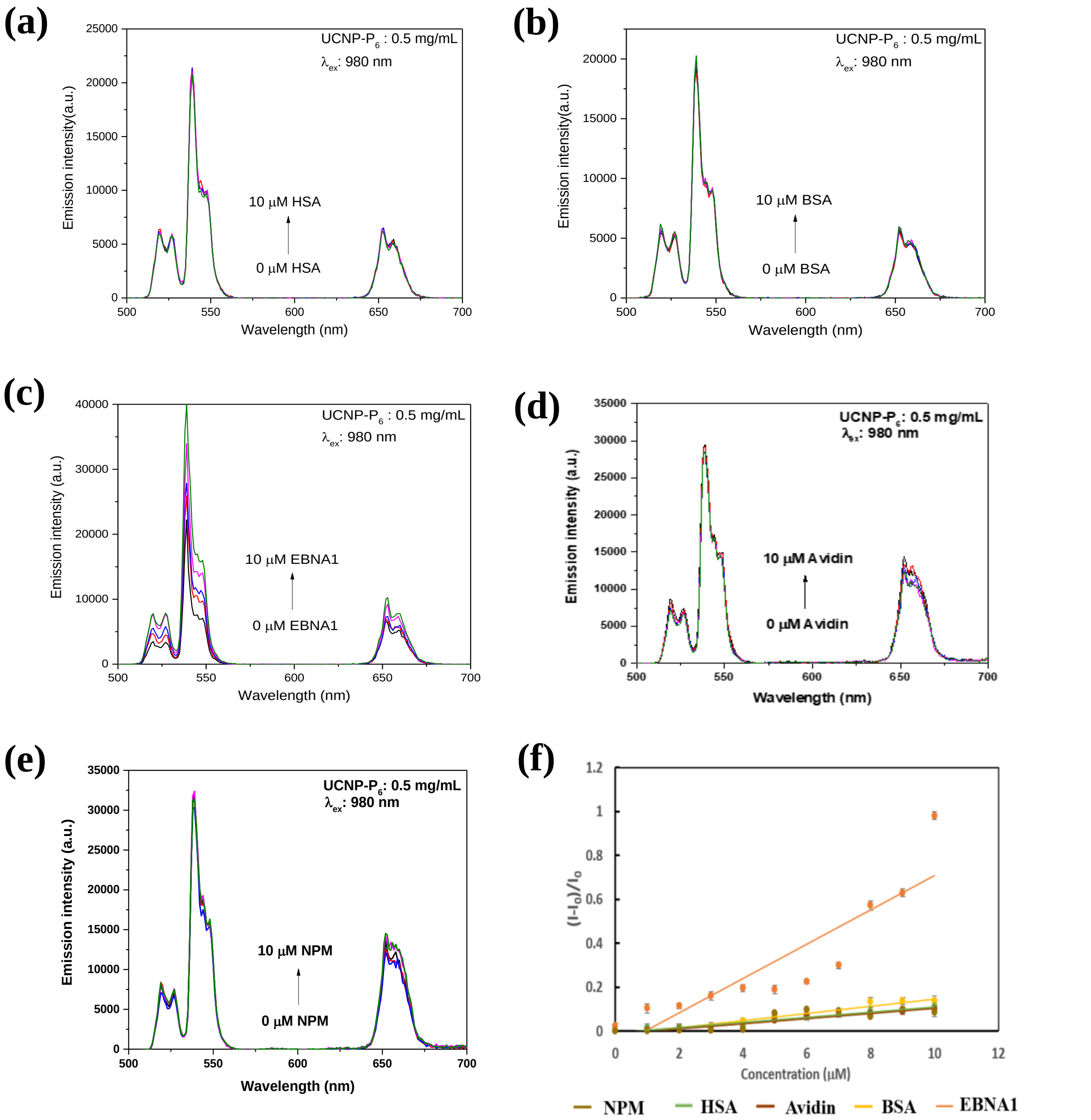

Figure S20. Luminescence titration of UCNP-P_6 (conc.: 0.5 mg/mL; excitation at 980 nm) towards a) HSA, b) BSA, c) EBNA1, d) Avidin, e) NPM and f) change in emission intensity of UCNP-P_6 on addition of NPM, HSA, Avidin, BSA and EBNA1.

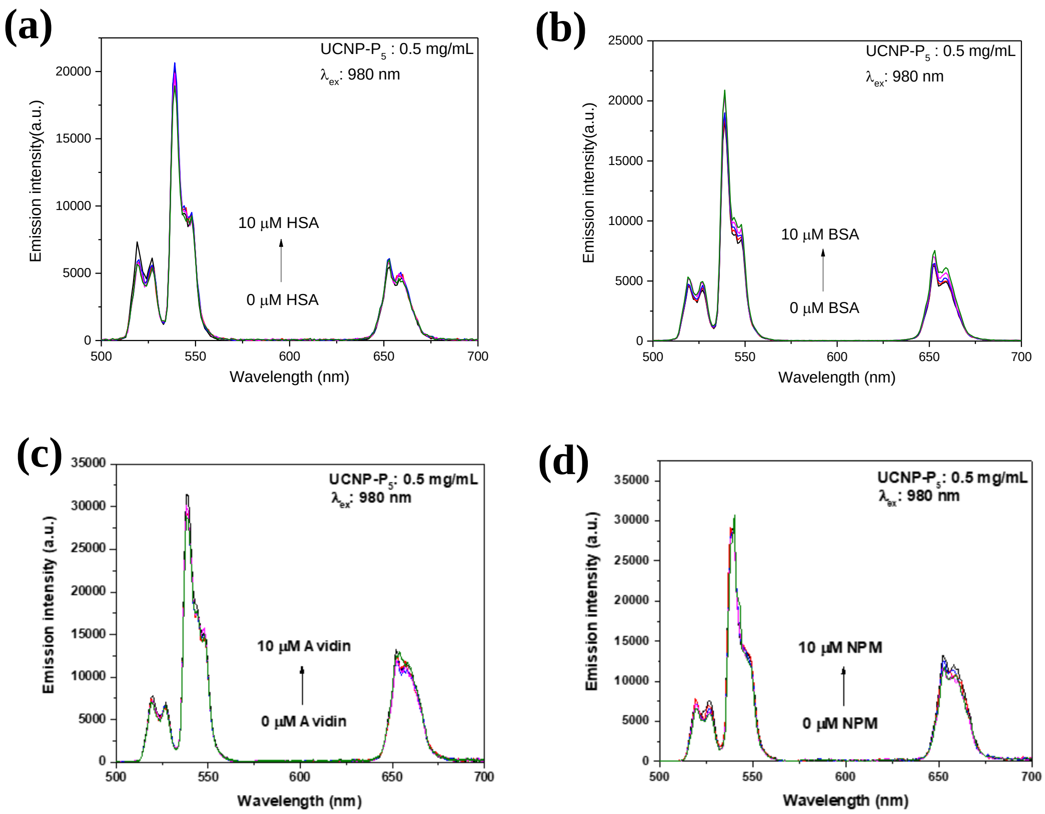

Figure S21. Luminescence titration of UCNP-P_5 (conc.: 0.5 mg/mL; excitation at 980 nm) towards a) HSA, b) BSA, c) Avidin and d) NPM.

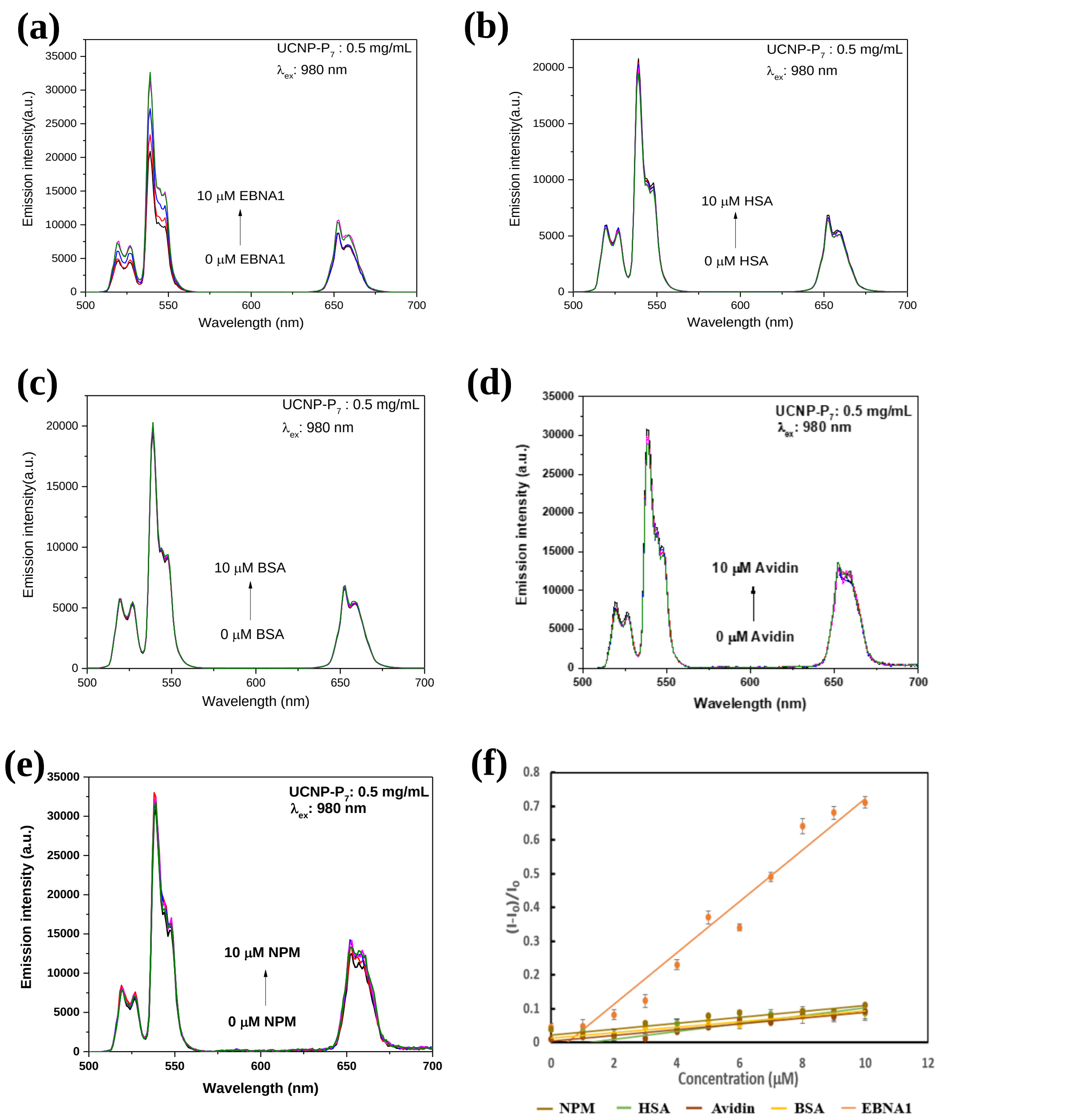

Figure S22. Luminescence titration of UCNP-P_7 (conc.: 0.5 mg/mL; excitation at 980 nm) towards a) EBNA1, b) HSA, c) BSA, d) Avidin, e) NPM and f) change in emission intensity of UCNP on addition of NPM, HSA, Avidin, BSA and EBNA1.

Table S3. Summary on all cell lines used in the work.

	EBNA1(+)	LMP1(+)	Cell lines
EBV(+) cells	√[a)]	√√	LCL3, Raji
	√√[b)]	√	C666, NPC43

EBV(-) cells	x[c)]	√√	HK1-LMP1
	x	x	HK1, HeLa, MRC-5

[a)](Positive); [b)](Highly positive); [c)](Negative)

(a) (b)

(c) (d)

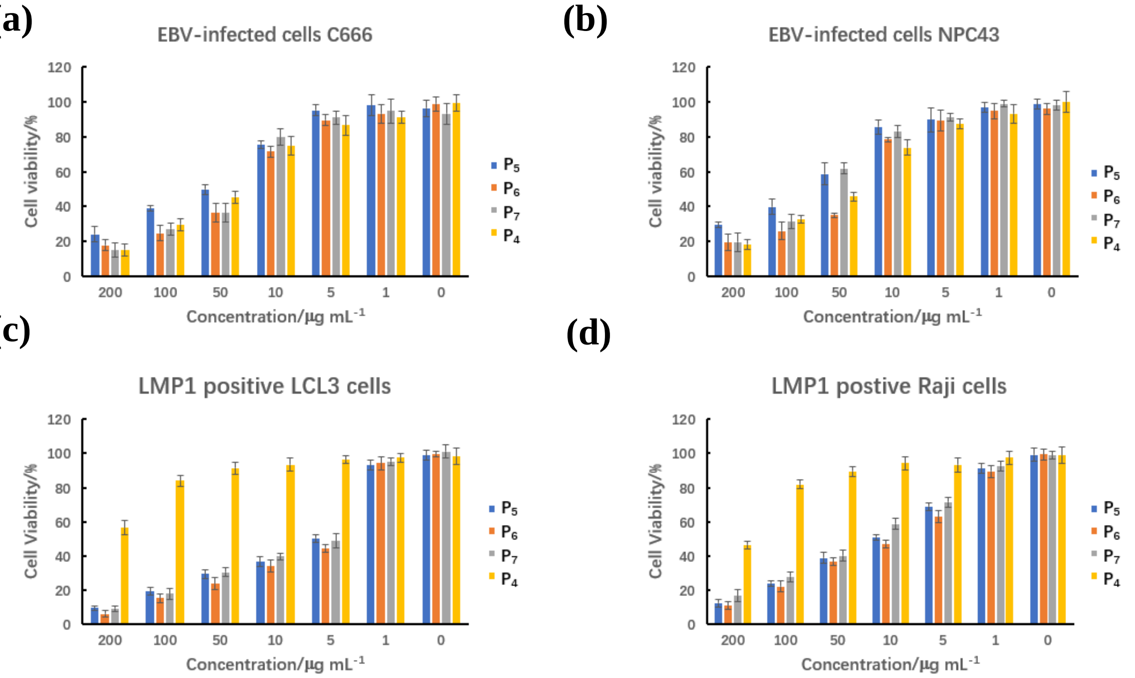

Figure S23. Cytotoxicity assay of P_5, P_6, P_7 and P_4 on (a) EBV-positive nasopharyngeal carcinoma C666, (b) EBV-positive nasopharyngeal carcinoma NPC43, (c) LMP1 positive LCL3 cells and (d) LMP1 positive Raji cells were assayed (incubation time: 24 hours). P_4: -AhxYFMVFGGRrRK

(a) (b)

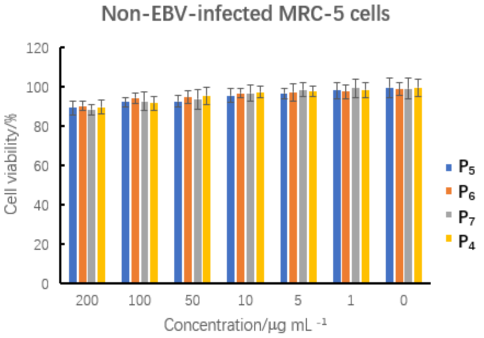

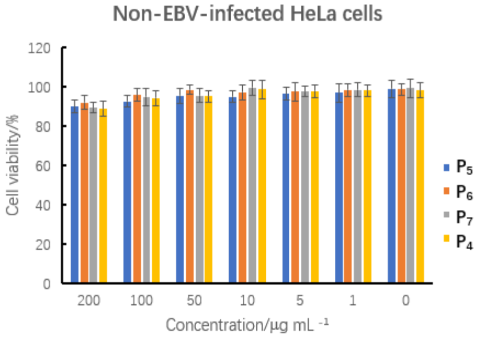

(c)

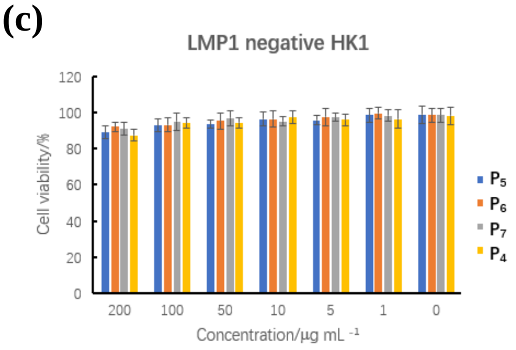

(d)

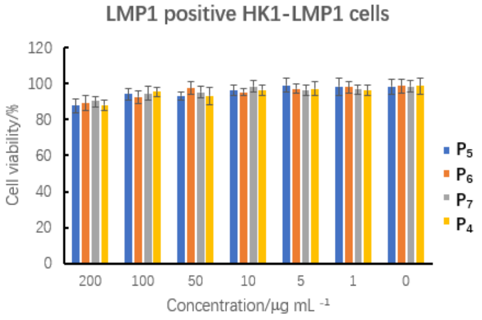

Figure S24. Cytotoxicity assay of P_5, P_6, P_7 and P_4 on (a) EBV-negative human lung fibroblast normal MRC-5 cells, (b) EBV-negative human cervical carcinoma HeLa, (c) LMP1 negative HK1 cells and (d) LMP1 positive HK1-LMP1 cells were assayed (incubation time: 24 hours). P_4: -AhxYFMVFGGRrRK

(a) **(b)**

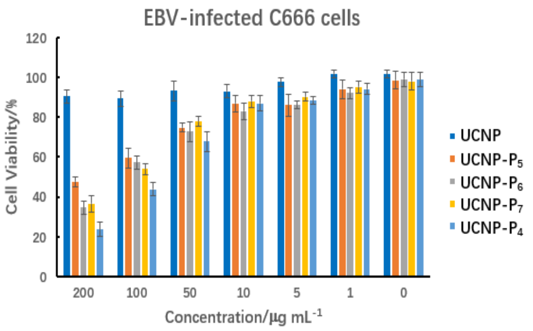

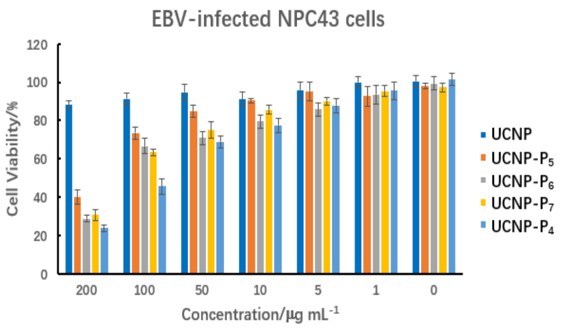

(c)

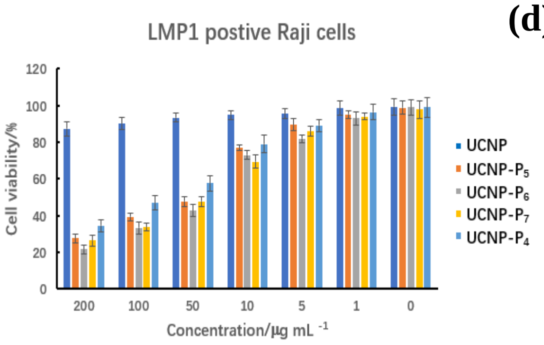

(d)

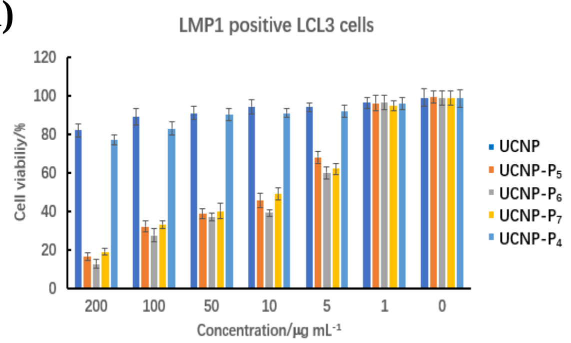

(e)

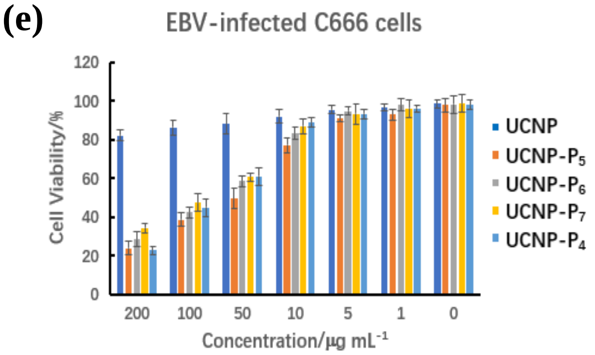

Figure S25. Cytotoxicity assay of UCNP, UCNP-P_n (n=5,6 and 7), UCNP-P_4 on (a) EBV-infected C666 cells, (b) EBV-infected NPC43 cells, (c) LMP1 positive Raji cells and (d) LMP1 positive LCL3 cells were assayed (incubation time: 24 hours). (e) EBV-infected C666 cells (incubation time: 5 days)

(a) **(b)**

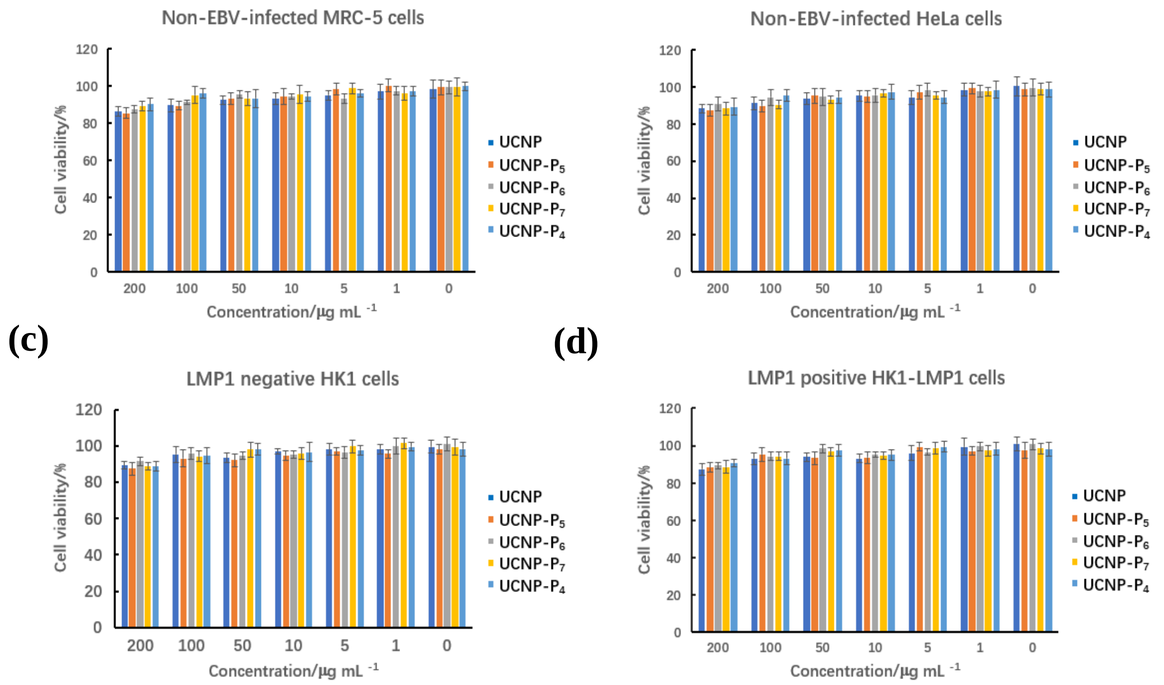

Figure S26. Cytotoxicity assay of UCNP, UCNP-P$_n$ (n=5,6 and 7), UCNP-P$_4$ on (a) EBV-negative MRC-5 cells, (b) EBV-negative HeLa cells, (c) LMP1 negative HK1 cells and (d) LMP1 positive HK1-LMP1 cells were assayed (incubation time: 24 hours).

Table S4. Summary on IC$_{50}$ (half inhibitory concentration) values of UCNP, UCNP-P$_n$ (n=5,6 and 7) and UCNP-P$_4$ in C666, LCL3, NPC43 and Raji cells for 24 h incubation time and 5-day incubation time respectively.

	IC$_{50}$ C666		IC$_{50}$ LCL3		IC$_{50}$ NPC43		IC$_{50}$ Raji	
UCNP	947[a)]	626[b)]	1024	585	879	462	963	441
UCNP-P$_5$	69	38	36	22	52	36	58	28
UCNP-P$_6$	37	41	19	21	38	34	27	29
UCNP-P$_7$	49	54	28	29	61	49	55	39
UCNP-P$_4$	58	51	443	361	73	47	61	52

[a)](IC$_{50}$ values for 24 h incubation time in black); [b)](IC$_{50}$ values for 5-day incubation time in red).

(a)

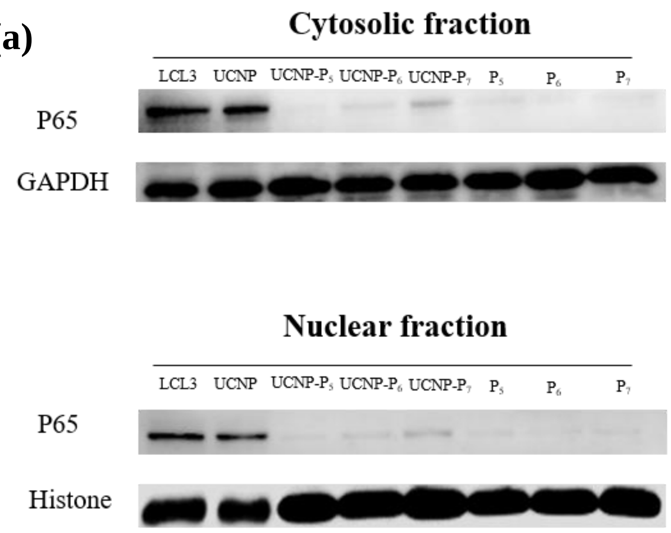

(b)

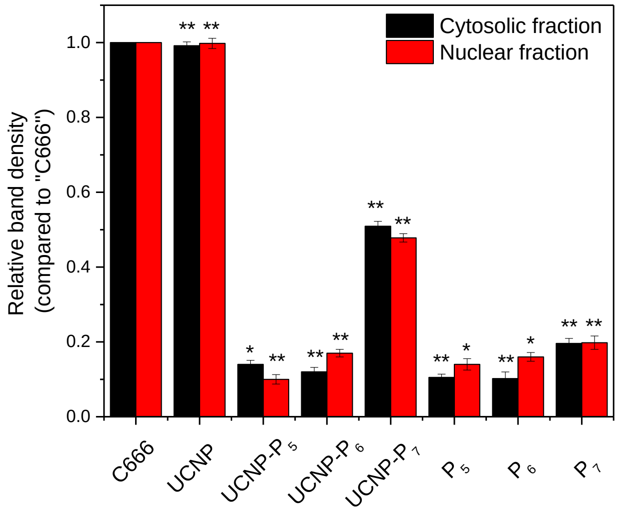

Figure S27. (a) Western blotting of UCNP and UCNP-P_n (n=5, 6 and 7) in cytosolic and nuclear fraction in C666 cells; (b) Quantitative analysis of p65 protein expression level in cytosolic and nuclear fraction in C666 cells after various treatments. *P < 0.05, **P < 0.01.

(a)

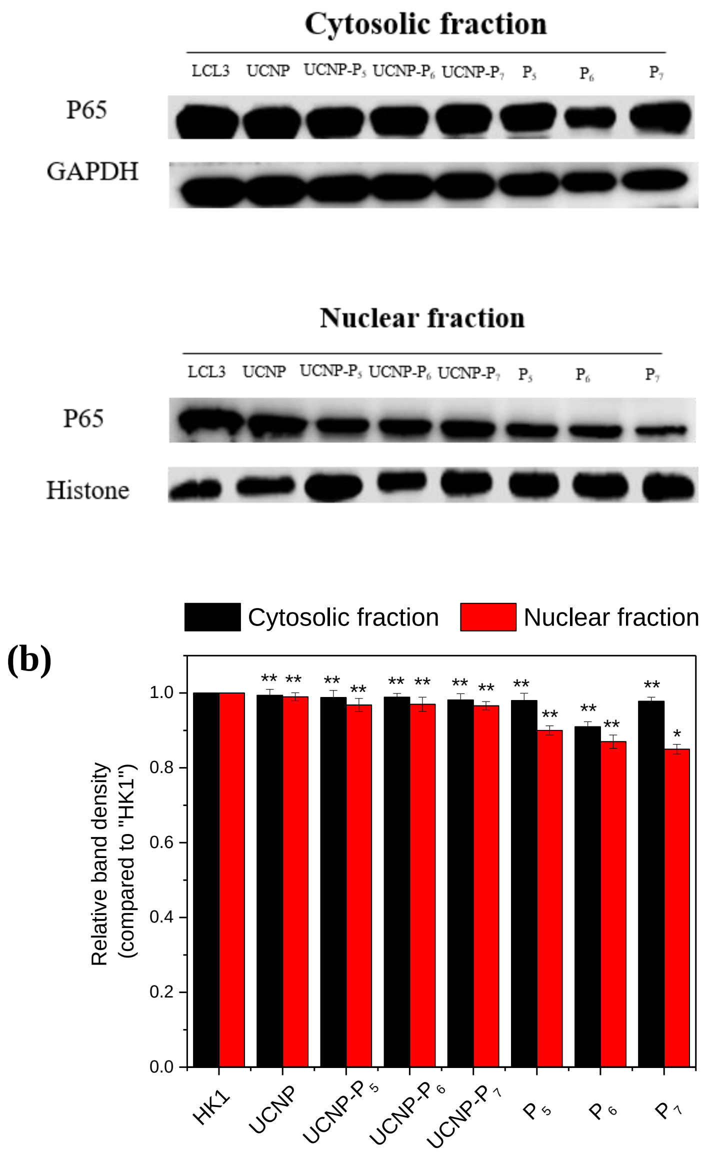

Figure S28. (a) Western blotting of UCNP and UCNP-P_n (n=5, 6 and 7) in cytosolic and nuclear fraction in HK1 cells; (b) Quantitative analysis of p65 protein expression level in cytosolic and nuclear fraction in HK1 cells after various treatments. *$P < 0.05$, **$P < 0.01$.

(u)

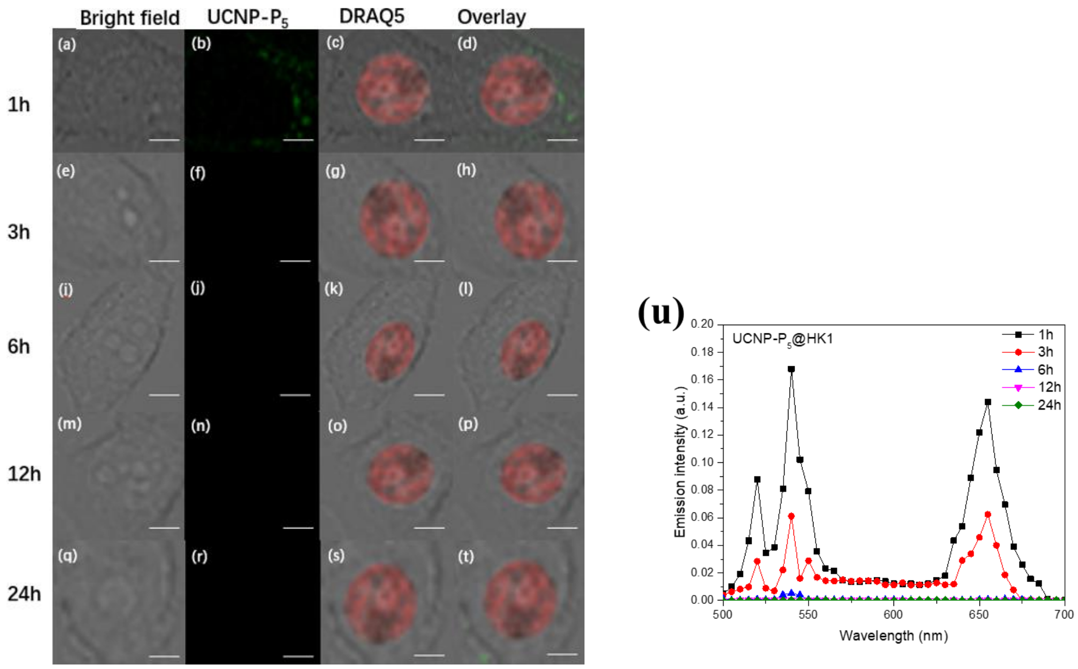

Figure S29. Two-photon confocal images of UCNP-P_5 in EBV-negative HK1 cells (λ_{ex}=980 nm, λ_{em}=500-700 nm); (a)-(d): bright field, UCNP-P_5 treated with HK1 cells for 1 h, DRAQ5 fluorescence and overlay image respectively; (e)-(h): bright field, UCNP-P_5 treated with HK1 cells for 3 h, DRAQ5 fluorescence and overlay image respectively; (i)-(l): bright field, UCNP-P_5 treated with HK1 cells for 6 h, DRAQ5 fluorescence and overlay image respectively; (m)-(p): bright field, UCNP-P_5 treated with HK1 cells for 12 h, DRAQ5 fluorescence and overlay image respectively; (q)-(t): bright field, UCNP-P_5 treated with HK1 cells for 24 h, DRAQ5 fluorescence and overlay image respectively; (u) Lambda scan of UCNP-P_5 in EBV-negative HK1 cells in different time intervals of 1 h, 3 h, 6 h, 12 h and 24 h.

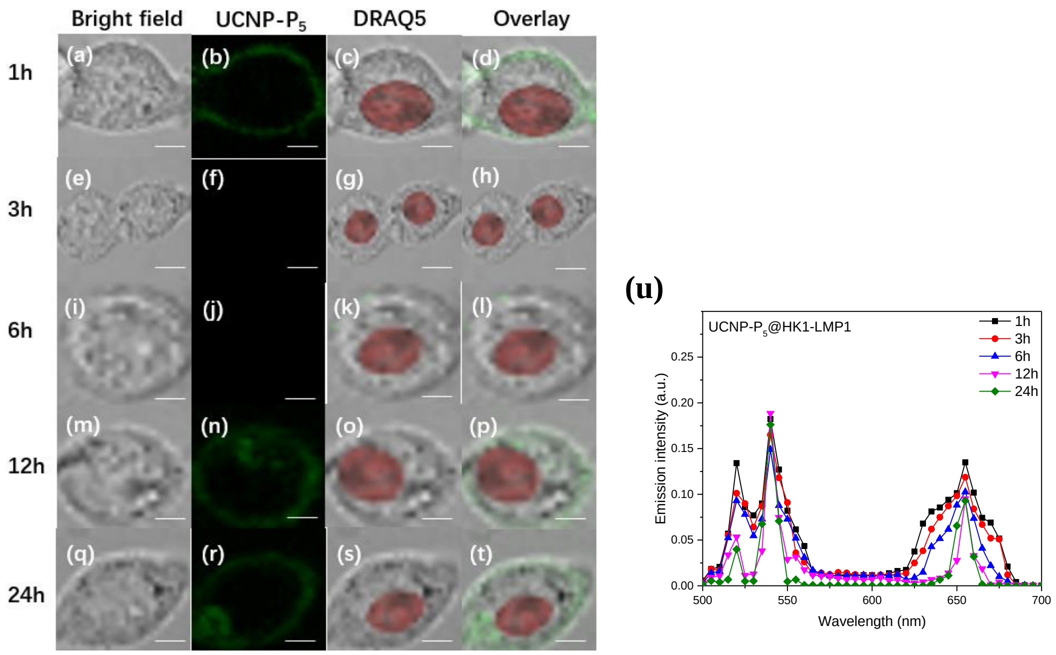

Figure S30. Two-photon confocal images of UCNP-P_5 in EBV-negative HK1-LMP1 cells (λ_{ex}=980 nm, λ_{em}=500-700 nm); (a)-(d): bright field, UCNP-P_5 treated with HK1-LMP1 cells for 1 h, DRAQ5 fluorescence and overlay image respectively; (e)-(h): bright field, UCNP-P_5 treated with HK1-LMP1 cells for 3 h, DRAQ5 fluorescence and overlay image respectively; (i)-(l): bright field, UCNP-P_5 treated with HK1-LMP1 cells for 6 h, DRAQ5 fluorescence and overlay image respectively; (m)-(p): bright field, UCNP-P_5 treated with HK1-LMP1 cells for 12 h, DRAQ5 fluorescence and overlay image respectively; (q)-(t): bright field, UCNP-P_5 treated with HK1-LMP1 cells for 24 h, DRAQ5 fluorescence and overlay image respectively; (u) Lambda scan of UCNP-P_5 in EBV-negative HK1-LMP1 cells in different time intervals of 1 h, 3 h, 6 h, 12 h and 24 h.

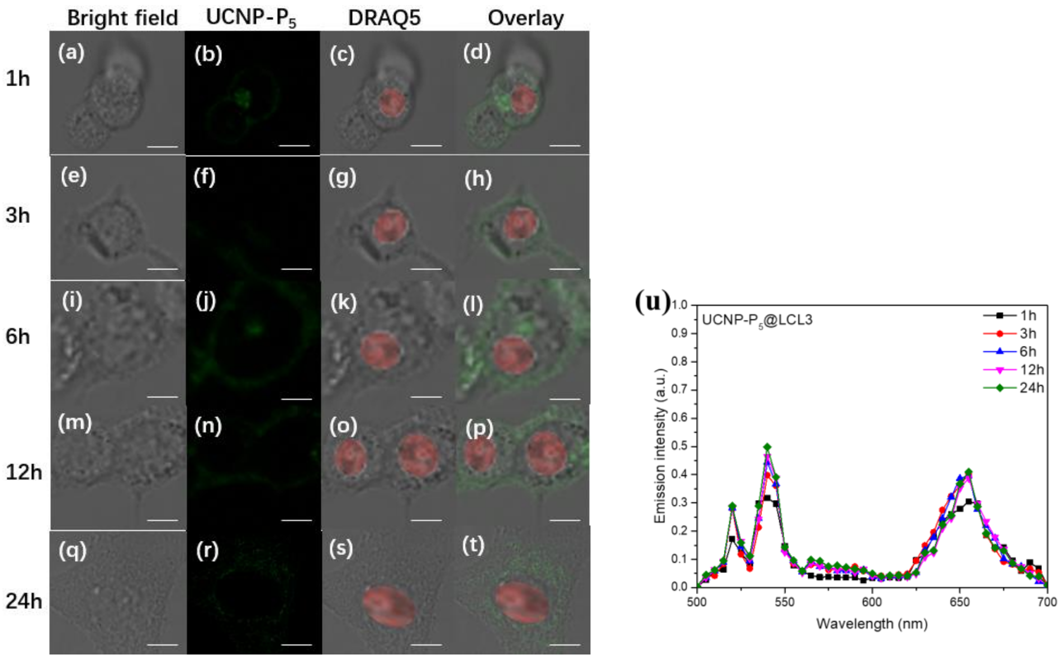

Figure S31. Two-photon confocal images of UCNP-P$_5$ in EBV-positive LCL3 cells (λ_{ex}=980 nm, λ_{em}=500-700 nm); (a)-(d): bright field, UCNP-P$_5$ treated with LCL3 cells for 1 h, DRAQ5 fluorescence and overlay image respectively; (e)-(h): bright field, UCNP-P$_5$ treated with LCL3 cells for 3 h, DRAQ5 fluorescence and overlay image respectively; (i)-(l): bright field, UCNP-P$_5$ treated with LCL3 cells for 6 h, DRAQ5 fluorescence and overlay image respectively; (m)-(p): bright field, UCNP-P$_5$ treated with LCL3 cells for 12 h, DRAQ5 fluorescence and overlay image respectively; (q)-(t): bright field, UCNP-P$_5$ treated with LCL3 cells for 24 h, DRAQ5 fluorescence and overlay image respectively; (u) Lambda scan of UCNP-P$_5$ in EBV-positive LCL3 cells in different time intervals of 1 h, 3 h, 6 h, 12 h and 24 h.

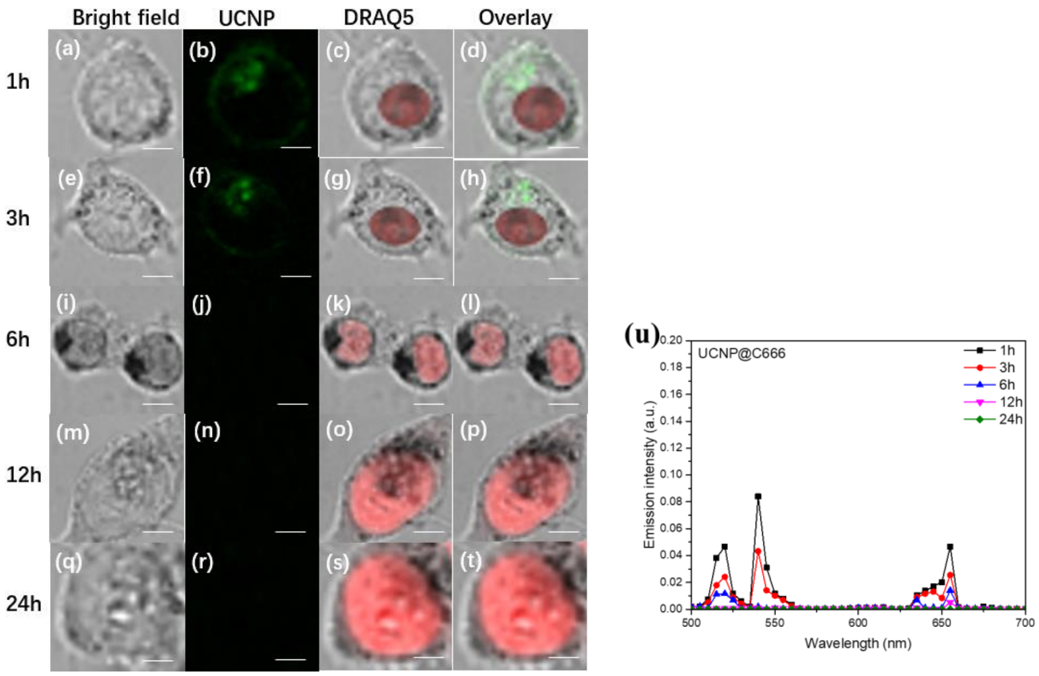

Figure S32. Two-photon confocal images of UCNP in EBV-positive C666 cells (λ_{ex}=980 nm, λ_{em}=500-700 nm); (a)-(d): bright field, UCNP treated with C666 cells for 1 h, DRAQ5 fluorescence and overlay image respectively; (e)-(h): bright field, UCNP treated with C666 cells for 3 h, DRAQ5 fluorescence and overlay image respectively; (i)-(l): bright field, UCNP treated with C666 cells for 6 h, DRAQ5 fluorescence and overlay image respectively; (m)-(p): bright field, UCNP treated with C666 cells for 12 h, DRAQ5 fluorescence and overlay image respectively; (q)-(t): bright field, UCNP treated with C666 cells for 24 h, DRAQ5 fluorescence and overlay image respectively; (u) Lambda scan of UCNP in EBV-positive C666 cells in different time intervals of 1 h, 3 h, 6 h, 12 h and 24 h.

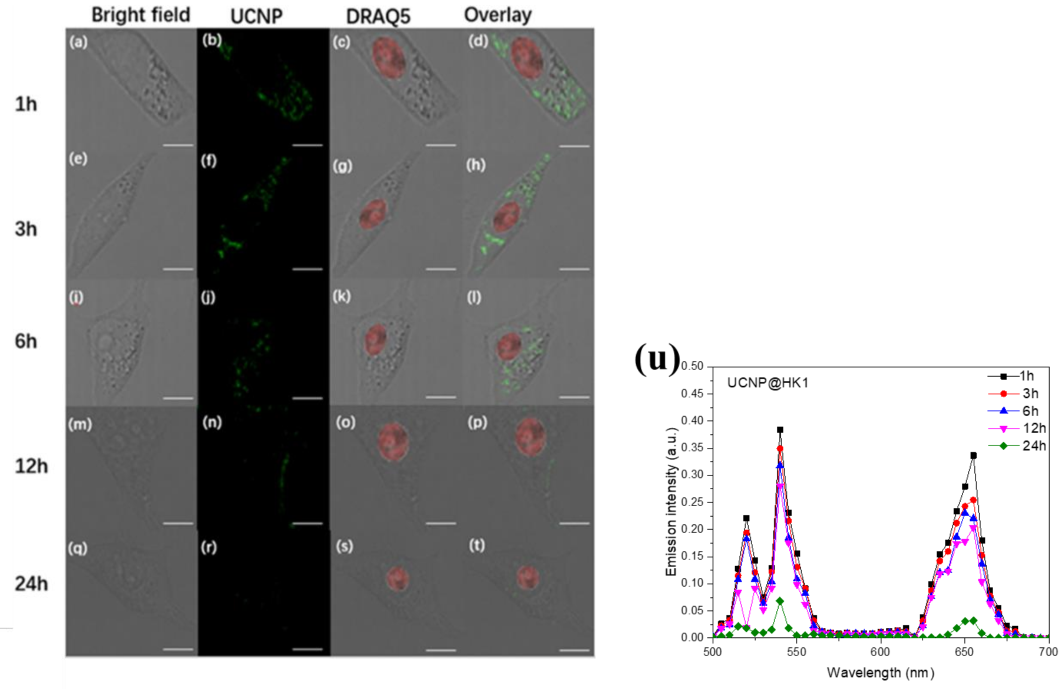

Figure S33. Two-photon confocal images of UCNP in EBV-negative HK1 cells (λ_{ex}=980 nm, λ_{em}=500-700 nm); (a)-(d): bright field, UCNP treated with HK1 cells for 1 h, DRAQ5 fluorescence and overlay image respectively; (e)-(h): bright field, UCNP treated with HK1 cells for 3 h, DRAQ5 fluorescence and overlay image respectively; (i)-(l): bright field, UCNP treated with HK1 cells for 6 h, DRAQ5 fluorescence and overlay image respectively; (m)-(p): bright field, UCNP treated with HK1 cells for 12 h, DRAQ5 fluorescence and overlay image respectively; (q)-(t): bright field, UCNP treated with HK1 cells for 24 h, DRAQ5 fluorescence and overlay image respectively; (u) Lambda scan of UCNP in EBV-negative HK1 cells in different time intervals of 1 h, 3 h, 6 h, 12 h and 24 h.

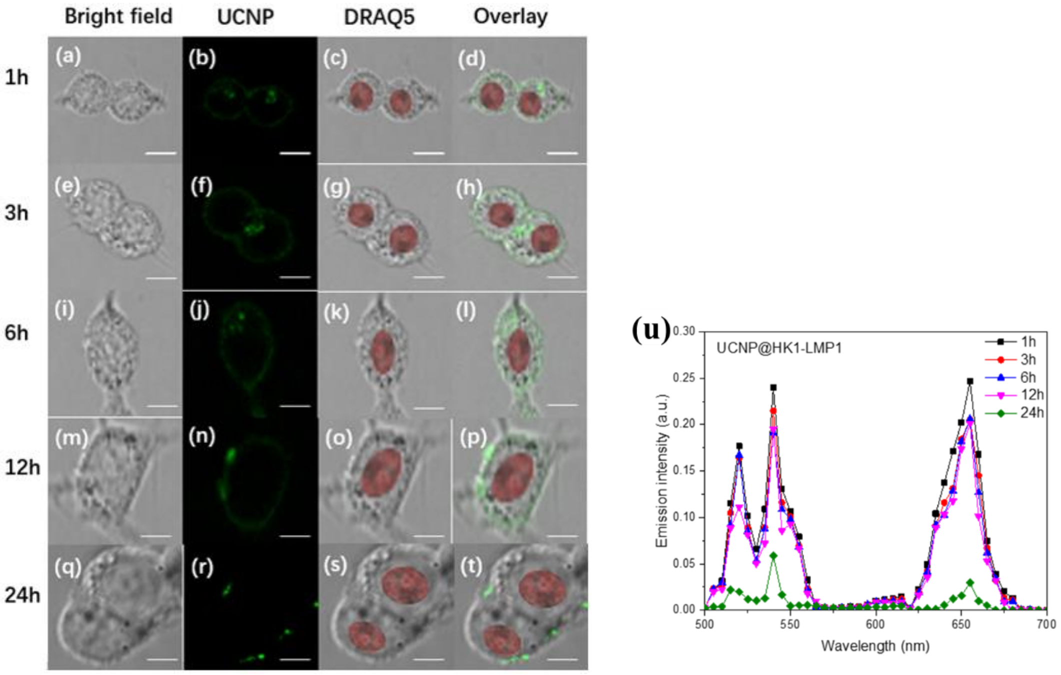

Figure S34. Two-photon confocal images of UCNP in EBV-negative HK1-LMP1 cells (λ_{ex}=980 nm, λ_{em}=500-700 nm); (a)-(d): bright field, UCNP treated with HK1-LMP1 cells for 1 h, DRAQ5 fluorescence and overlay image respectively; (e)-(h): bright field, UCNP treated with HK1-LMP1 cells for 3 h, DRAQ5 fluorescence and overlay image respectively; (i)-(l): bright field, UCNP treated with HK1-LMP1 cells for 6 h, DRAQ5 fluorescence and overlay image respectively; (m)-(p): bright field, UCNP treated with HK1-LMP1 cells for 12 h, DRAQ5 fluorescence and overlay image respectively; (q)-(t): bright field, UCNP treated with HK1-LMP1 cells for 24 h, DRAQ5 fluorescence and overlay image respectively; (u) Lambda scan of UCNP in EBV-negative HK1-LMP1 cells in different time intervals of 1 h, 3 h, 6 h, 12 h and 24 h.

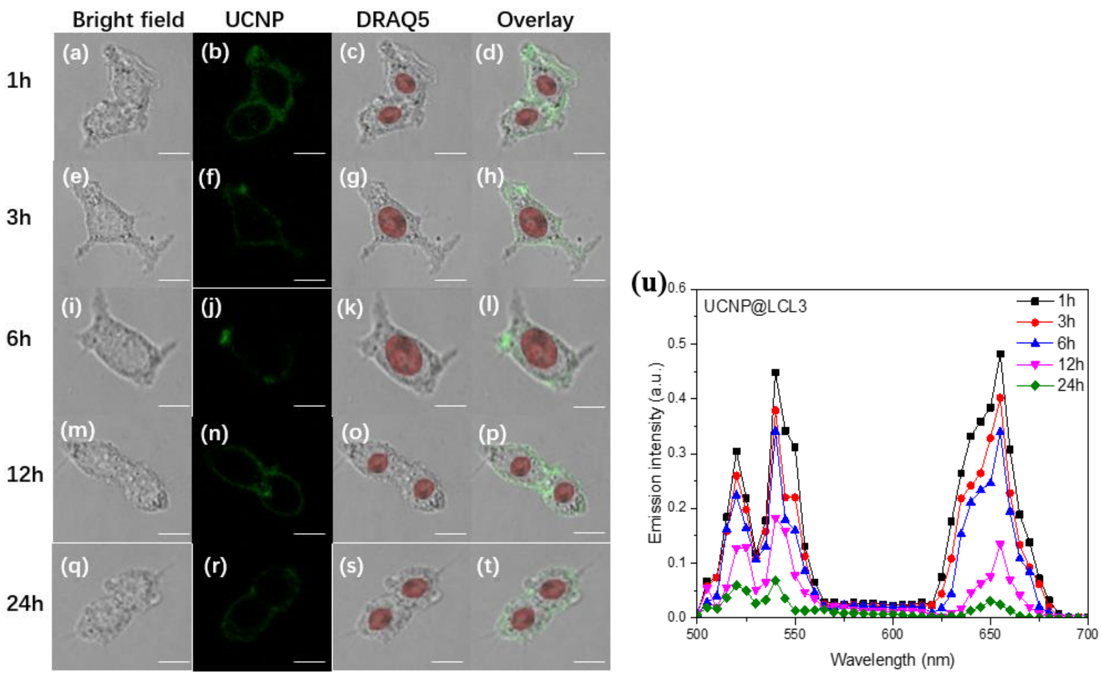

Figure S35. Two-photon confocal images of UCNP in EBV-positive LCL3 cells (λ_{ex}=980 nm, λ_{em}=500-700 nm); (a)-(d): bright field, UCNP treated with LCL3 cells for 1 h, DRAQ5 fluorescence and overlay image respectively; (e)-(h): bright field, UCNP treated with LCL3 cells for 3 h, DRAQ5 fluorescence and overlay image respectively; (i)-(l): bright field, UCNP treated with LCL3 cells for 6 h, DRAQ5 fluorescence and overlay image respectively; (m)-(p): bright field, UCNP treated with LCL3 cells for 12 h, DRAQ5 fluorescence and overlay image respectively; (q)-(t): bright field, UCNP treated with LCL3 cells for 24 h, DRAQ5 fluorescence and overlay image respectively; (u) Lambda scan of UCNP in EBV-positive LCL3 cells in different time intervals of 1 h, 3 h, 6 h, 12 h and 24 h.

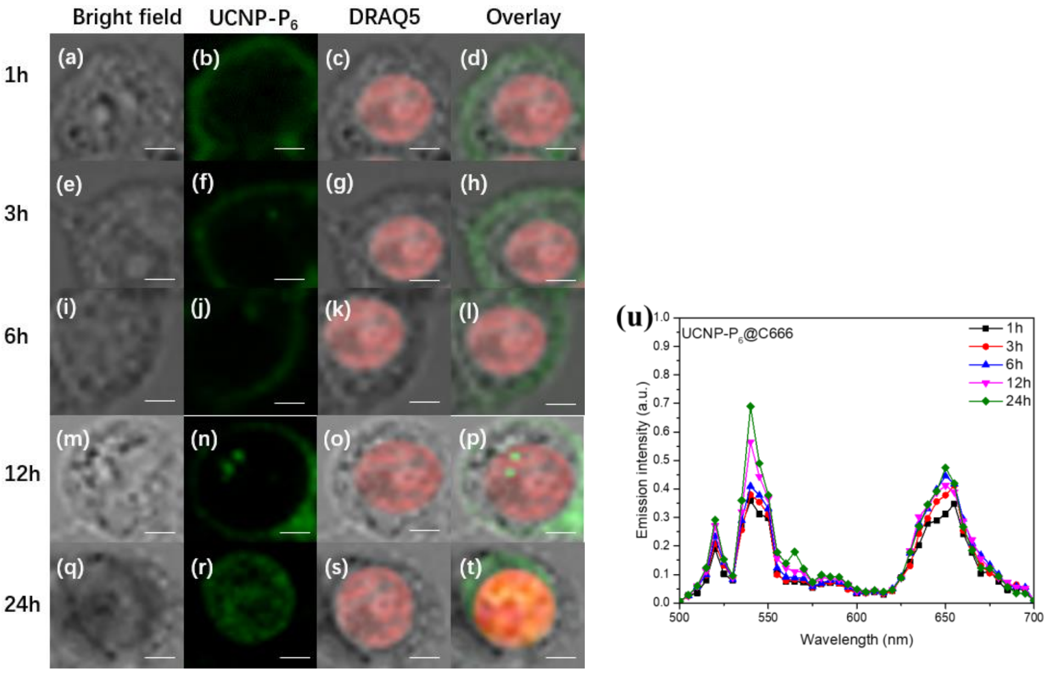

Figure S36. Two-photon confocal images of UCNP-P_6 in EBV-positive C666 cells (λ_{ex}=980 nm, λ_{em}=500-700 nm); (a)-(d): bright field, UCNP-P_6 treated with C666 cells for 1 h, DRAQ5 fluorescence and overlay image respectively; (e)-(h): bright field, UCNP-P_6 treated with C666 cells for 3 h, DRAQ5 fluorescence and overlay image respectively; (i)-(l): bright field, UCNP-P_6 treated with C666 cells for 6 h, DRAQ5 fluorescence and overlay image respectively; (m)-(p): bright field, UCNP-P_6 treated with C666 cells for 12 h, DRAQ5 fluorescence and overlay image respectively; (q)-(t): bright field, UCNP-P_6 treated with C666 cells for 24 h, DRAQ5 fluorescence and overlay image respectively; (u) Lambda scan of UCNP-P_6 in EBV-positive C666 cells in different time intervals of 1 h, 3 h, 6 h, 12 h and 24 h.

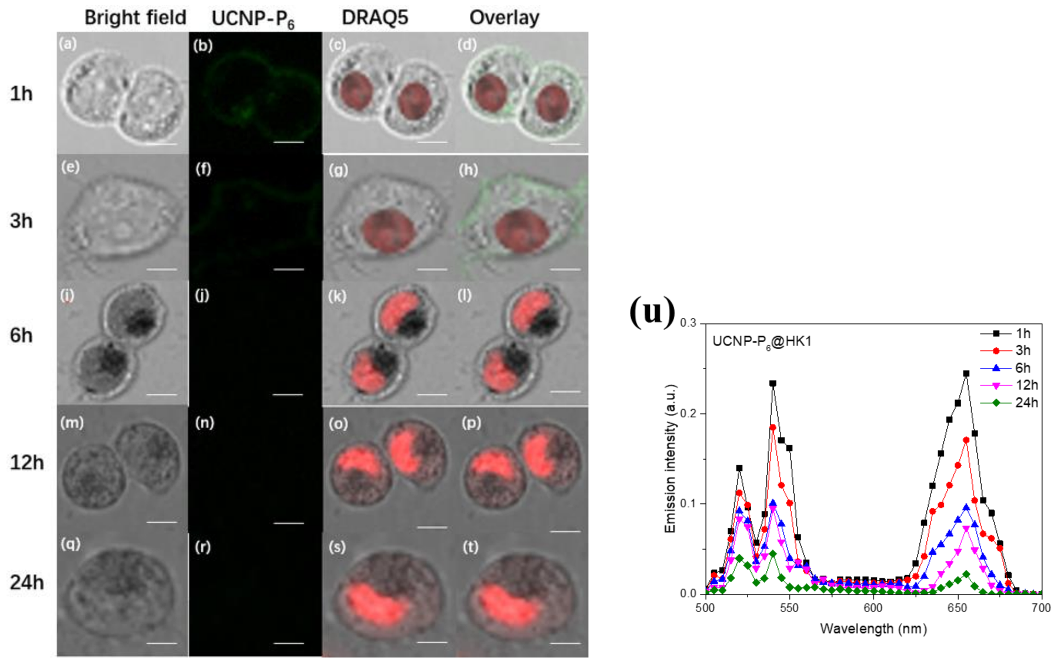

Figure S37. Two-photon confocal images of UCNP-P_6 in EBV-negative HK1 cells (λ_{ex}=980 nm, λ_{em}=500-700 nm); (a)-(d): bright field, UCNP-P_6 treated with HK1 cells for 1 h, DRAQ5 fluorescence and overlay image respectively; (e)-(h): bright field, UCNP-P_6 treated with HK1 cells for 3 h, DRAQ5 fluorescence and overlay image respectively; (i)-(l): bright field, UCNP-P_6 treated with HeLa cells for 6 h, DRAQ5 fluorescence and overlay image respectively; (m)-(p): bright field, UCNP-P_6 treated with HK1 cells for 12 h, DRAQ5 fluorescence and overlay image respectively; (q)-(t): bright field, UCNP-P_6 treated with HK1 cells for 24 h, DRAQ5 fluorescence and overlay image respectively; (u) Lambda scan of UCNP-P_6 in EBV-negative HK1 cells in different time intervals of 1 h, 3 h, 6 h, 12 h and 24 h.

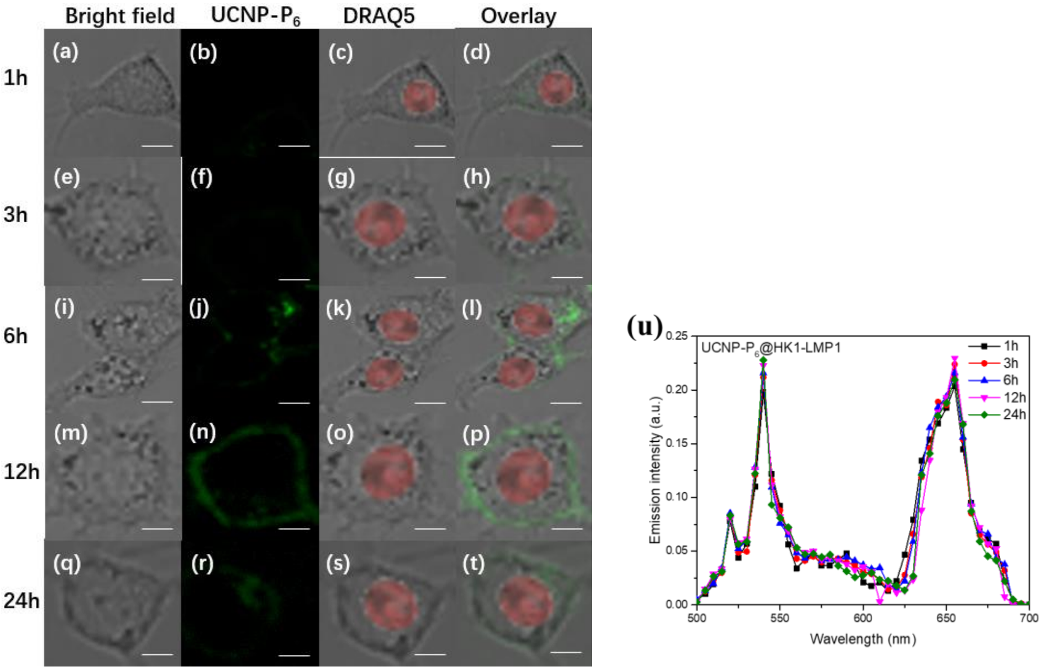

Figure S38. Two-photon confocal images of UCNP-P_6 in EBV-negative HK1-LMP1 cells (λ_{ex}=980 nm, λ_{em}=500-700 nm); (a)-(d): bright field, UCNP-P_6 treated with HK1-LMP1 cells for 1 h, DRAQ5 fluorescence and overlay image respectively; (e)-(h): bright field, UCNP-P_6 treated with HK1-LMP1 cells for 3 h, DRAQ5 fluorescence and overlay image respectively; (i)-(l): bright field, UCNP-P_6 treated with HK1-LMP1 cells for 6 h, DRAQ5 fluorescence and overlay image respectively; (m)-(p): bright field, UCNP-P_6 treated with HK1-LMP1 cells for 12 h, DRAQ5 fluorescence and overlay image respectively; (q)-(t): bright field, UCNP-P_6 treated with HK1-LMP1 cells for 24 h, DRAQ5 fluorescence and overlay image respectively; (u) Lambda scan of UCNP-P_6 in EBV-negative HK1-LMP1 cells in different time intervals of 1 h, 3 h, 6 h, 12 h and 24 h.

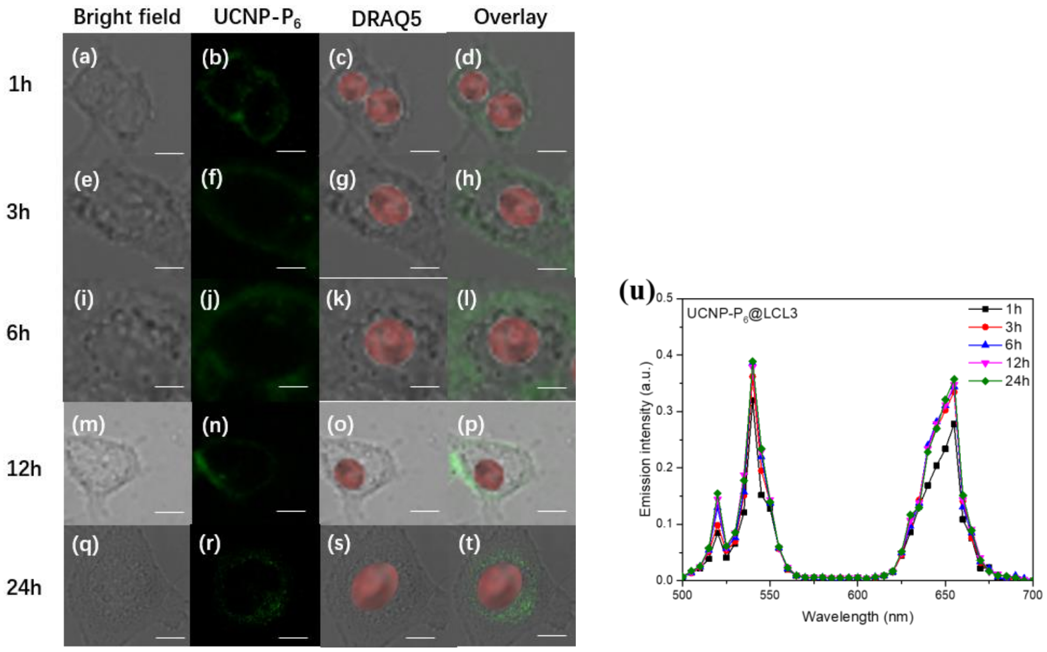

Figure S39. Two-photon confocal images of UCNP-P_6 in EBV-positive LCL3 cells (λ_{ex}=980 nm, λ_{em}=500-700 nm); (a)-(d): bright field, UCNP-P_6 treated with LCL3 cells for 1 h, DRAQ5 fluorescence and overlay image respectively; (e)-(h): bright field, UCNP-P_6 treated with LCL3 cells for 3 h, DRAQ5 fluorescence and overlay image respectively; (i)-(l): bright field, UCNP-P_6 treated with LCL3 cells for 6 h, DRAQ5 fluorescence and overlay image respectively; (m)-(p): bright field, UCNP-P_6 treated with LCL3 cells for 12 h, DRAQ5 fluorescence and overlay image respectively; (q)-(t): bright field, UCNP-P_6 treated with LCL3 cells for 24 h, DRAQ5 fluorescence and overlay image respectively; (u) Lambda scan of UCNP-P_6 in EBV-positive LCL3 cells in different time intervals of 1 h, 3 h, 6 h, 12 h and 24 h.

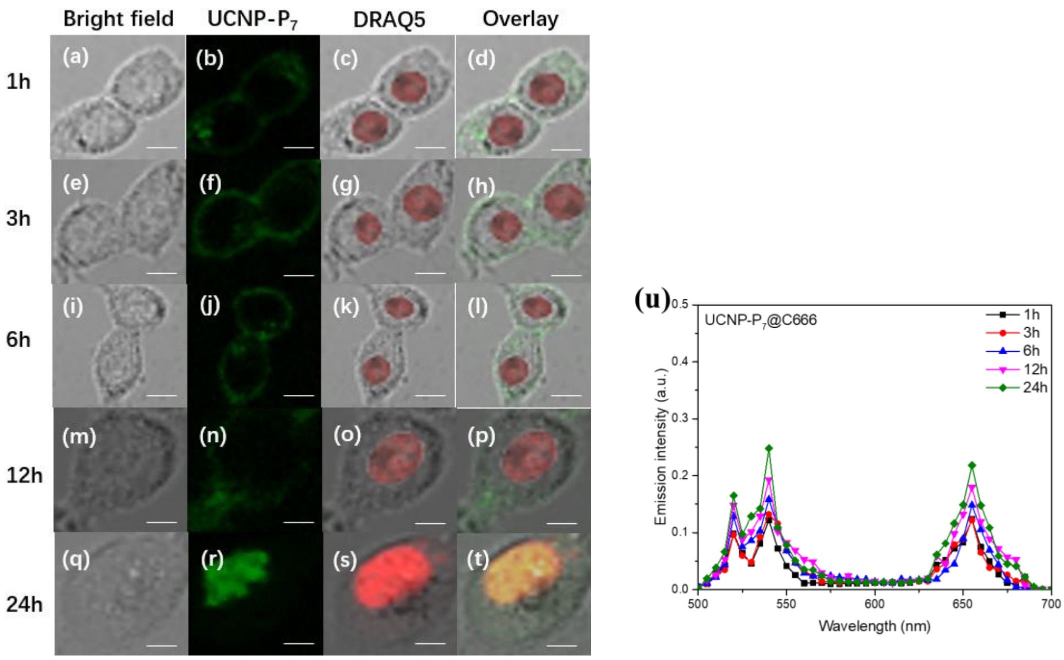

Figure S40. Two-photon confocal images of UCNP-P_7 in EBV-positive C666 cells (λ_{ex}=980 nm, λ_{em}=500-700 nm); (a)-(d): bright field, UCNP-P_7 treated with C666 cells for 1 h, DRAQ5 fluorescence and overlay image respectively; (e)-(h): bright field, UCNP-P_7 treated with C666 cells for 3 h, DRAQ5 fluorescence and overlay image respectively; (i)-(l): bright field, UCNP-P_7 treated with C666 cells for 6 h, DRAQ5 fluorescence and overlay image respectively; (m)-(p): bright field, UCNP-P_7 treated with C666 cells for 12 h, DRAQ5 fluorescence and overlay image respectively; (q)-(t): bright field, UCNP-P_7 treated with C666 cells for 24 h, DRAQ5 fluorescence and overlay image respectively; (u) Lambda scan of UCNP-P_7 in EBV-positive C666 cells in different time intervals of 1 h, 3 h, 6 h, 12 h and 24 h.

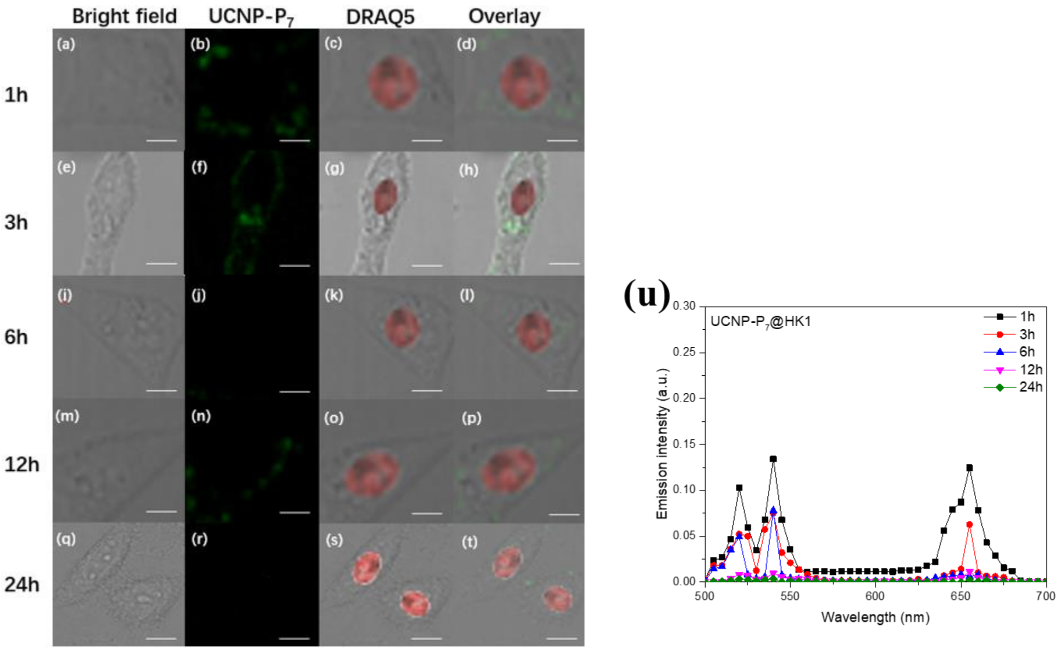

Figure S41. Two-photon confocal images of UCNP-P$_7$ in EBV-negative HK1 cells (λ_{ex}=980 nm, λ_{em}=500-700 nm); (a)-(d): bright field, UCNP-P$_7$ treated with HK1 cells for 1 h, DRAQ5 fluorescence and overlay image respectively; (e)-(h): bright field, UCNP-P$_7$ treated with HK1 cells for 3 h, DRAQ5 fluorescence and overlay image respectively; (i)-(l): bright field, UCNP-P$_7$ treated with HK1 cells for 6 h, DRAQ5 fluorescence and overlay image respectively; (m)-(p): bright field, UCNP-P$_7$ treated with HK1 cells for 12 h, DRAQ5 fluorescence and overlay image respectively; (q)-(t): bright field, UCNP-P$_7$ treated with HK1 cells for 24 h, DRAQ5 fluorescence and overlay image respectively; (u) Lambda scan of UCNP-P$_7$ in EBV-negative HK1 cells in different time intervals of 1 h, 3 h, 6 h, 12 h and 24 h.

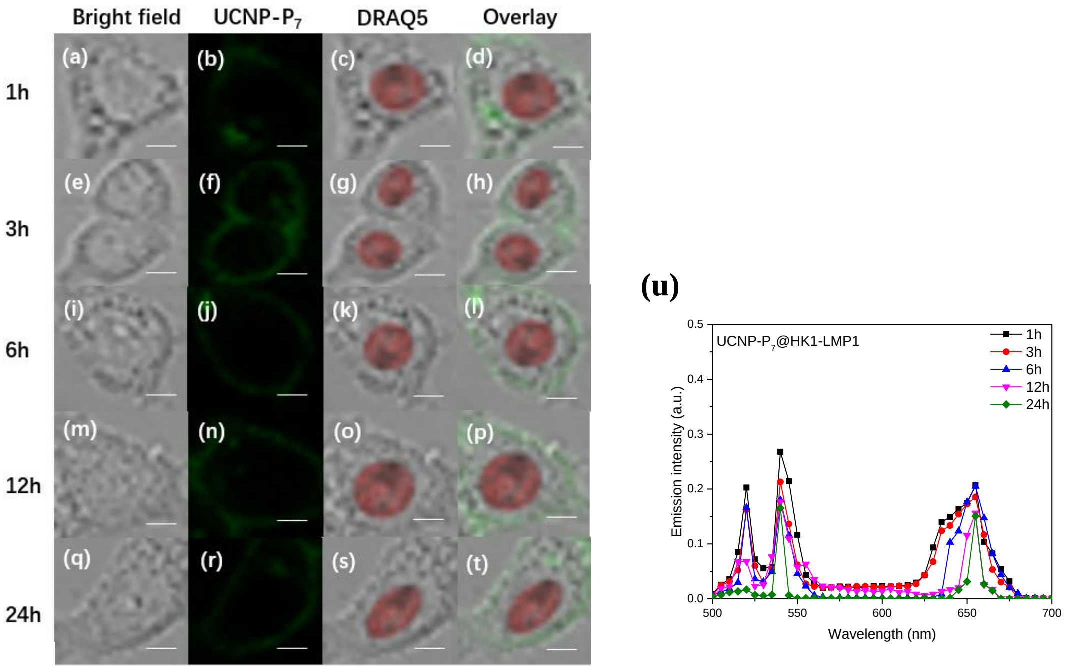

Figure S42. Two-photon confocal images of UCNP-P_7 in EBV-negative HK1-LMP1 cells (λ_{ex}=980 nm, λ_{em}=500-700 nm); (a)-(d): bright field, UCNP-P_7 treated with HK1-LMP1 cells for 1 h, DRAQ5 fluorescence and overlay image respectively; (e)-(h): bright field, UCNP-P_7 treated with HK1-LMP1 cells for 3 h, DRAQ5 fluorescence and overlay image respectively; (i)-(l): bright field, UCNP-P_7 treated with HK1-LMP1 cells for 6 h, DRAQ5 fluorescence and overlay image respectively; (m)-(p): bright field, UCNP-P_7 treated with HK1-LMP1 cells for 12 h, DRAQ5 fluorescence and overlay image respectively; (q)-(t): bright field, UCNP-P_7 treated with HK1-LMP1 cells for 24 h, DRAQ5 fluorescence and overlay image respectively; (u) Lambda scan of UCNP-P_7 in EBV-negative HK1-LMP1 cells in different time intervals of 1 h, 3 h, 6 h, 12 h and 24 h.

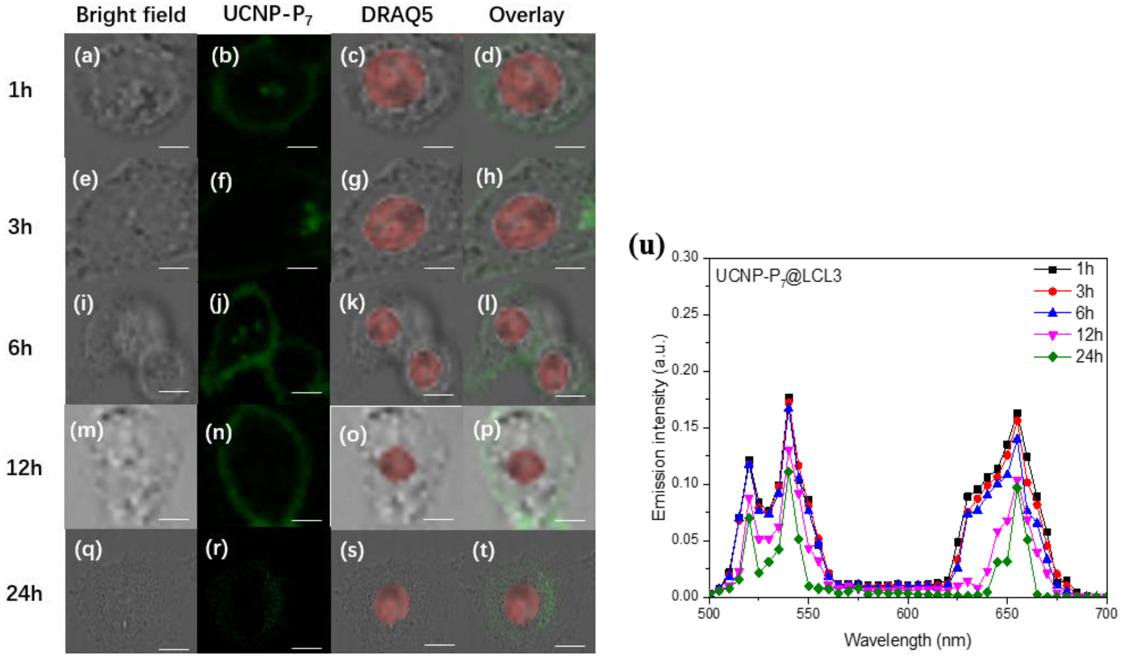

Figure S43. Two-photon confocal images of UCNP-P_7 in EBV-positive LCL3 cells (λ_{ex}=980 nm, λ_{em}=500-700 nm); (a)-(d): bright field, UCNP-P_7 treated with LCL3 cells for 1 h, DRAQ5 fluorescence and overlay image respectively; (e)-(h): bright field, UCNP-P_7 treated with LCL3 cells for 3 h, DRAQ5 fluorescence and overlay image respectively; (i)-(l): bright field, UCNP-P_7 treated with LCL3 cells for 6 h, DRAQ5 fluorescence and overlay image respectively; (m)-(p): bright field, UCNP-P_7 treated with LCL3 cells for 12 h, DRAQ5 fluorescence and overlay image respectively; (q)-(t): bright field, UCNP-P_7 treated with LCL3 cells for 24 h, DRAQ5 fluorescence and overlay image respectively; (u) Lambda scan of UCNP-P_7 in EBV-positive LCL3 cells in different time intervals of 1 h, 3 h, 6 h, 12 h and 24 h.

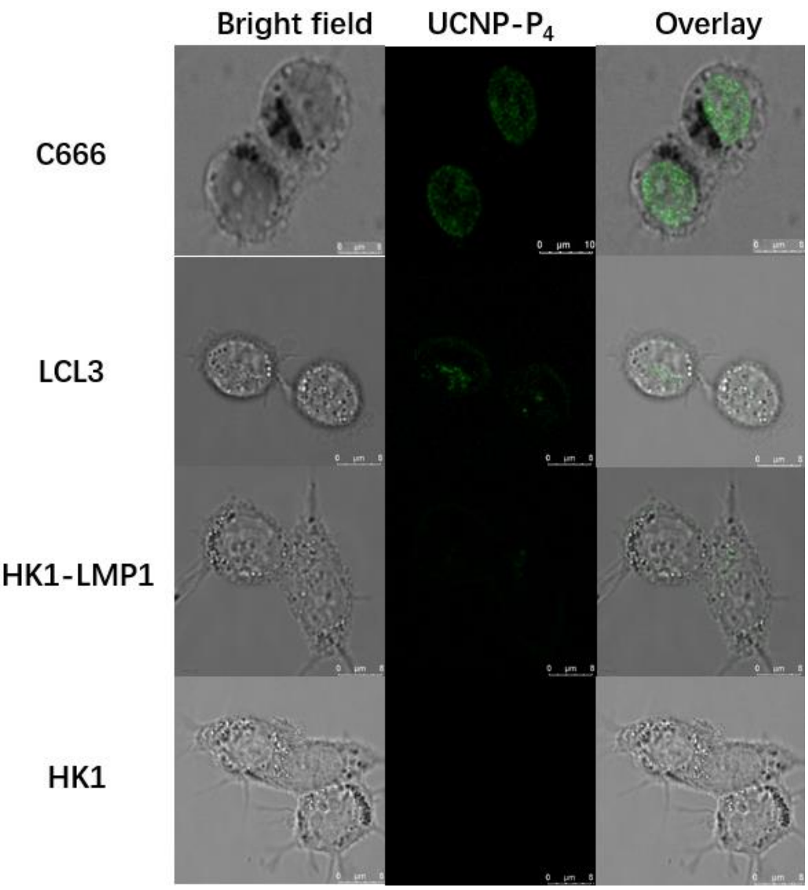

Figure S44. Two-photon confocal images of UCNP-P_4 in EBV-positive C666 cells, LMP1-positive LCL3 and HK1-LMP1 cells, EBV-negative HK1 cells. (λ_{ex}=980 nm, incubation time: 24 h)

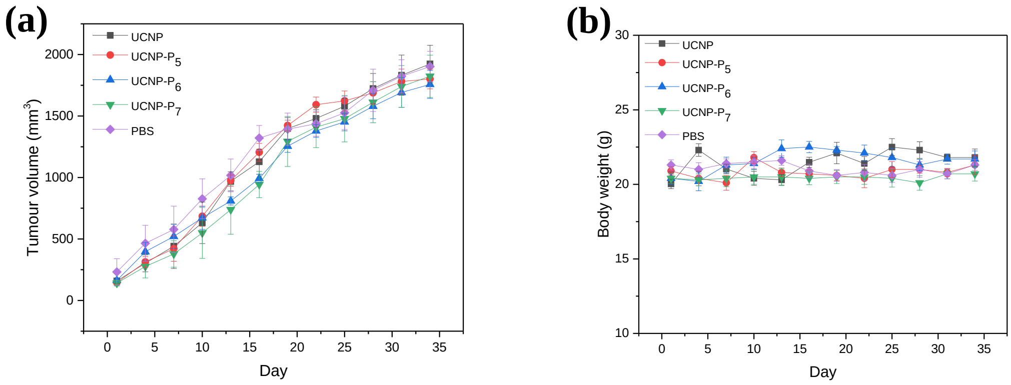

Figure S45. (a) Tumor volume of HeLa cell xenograft after treatment of UCNP, UCNP-P_5, UCNP-P_6, UCNP-P_7 and PBS during 33-day experimental period; (b) Body weight after treatment of UCNP, UCNP-P_5, UCNP-P_6, UCNP-P_7 and PBS.

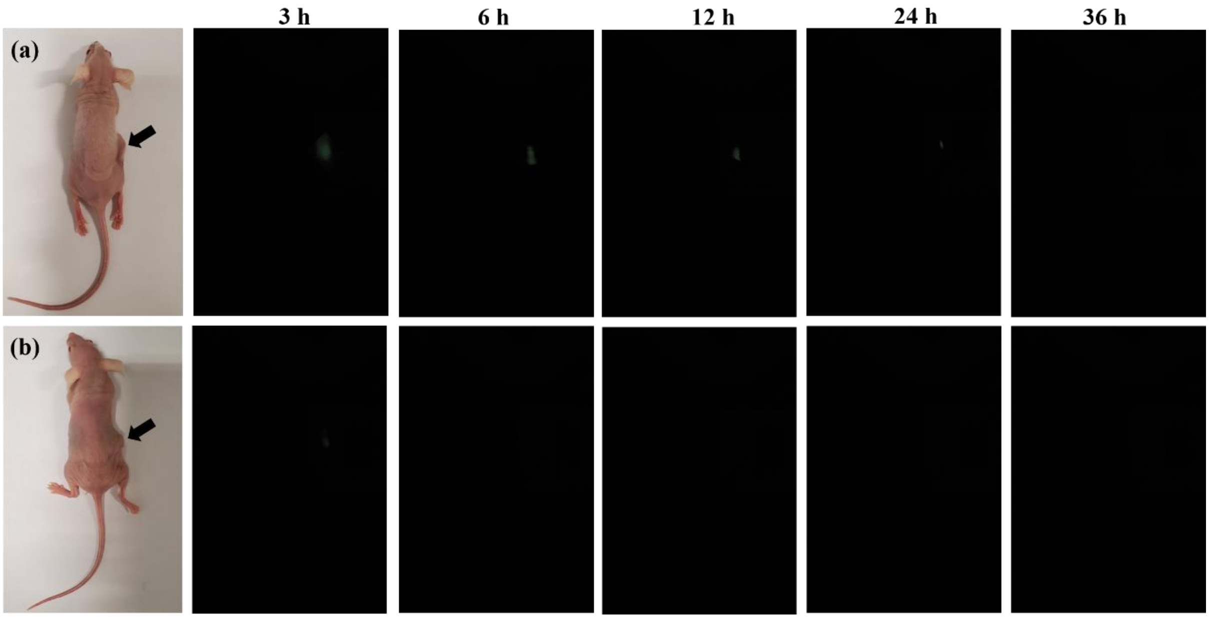

Figure S46. *In vivo* upconversion luminescence images of (a) C666-tumor-bearing (b) HeLa-tumor-bearing nude mice treating with UCNP-P_5 after various intravenous postinjection time at 3 h, 6 h, 12 h, 24 h and 36 h under 980 nm excitation. Arrows indicate the tumor sites.

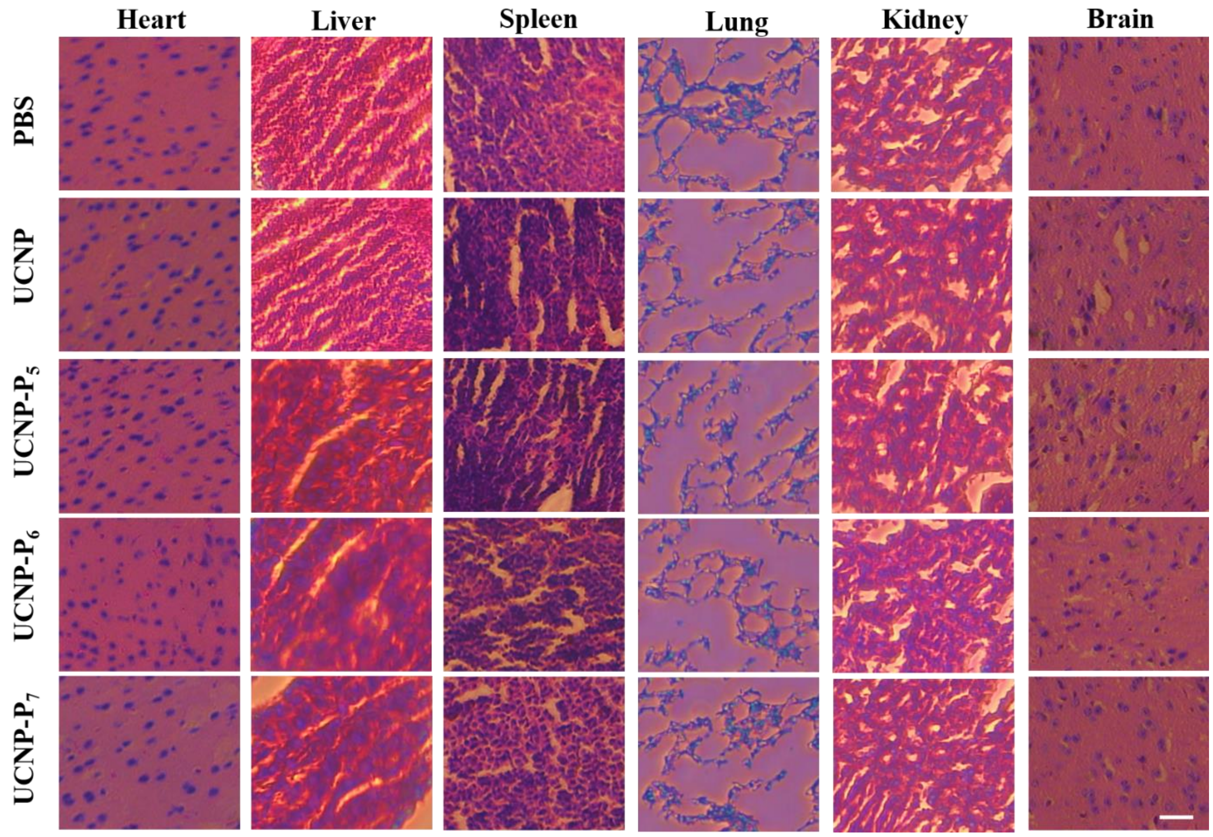

Figure S47. H&E staining of heart, liver, spleen, lung, kidney and brain from C666 tumor-bearing mice after different treatments including PBS, UCNP, UCNP-P_5, UCNP-P_6 and UCNP-P_7. Scale bar = 100 µm.

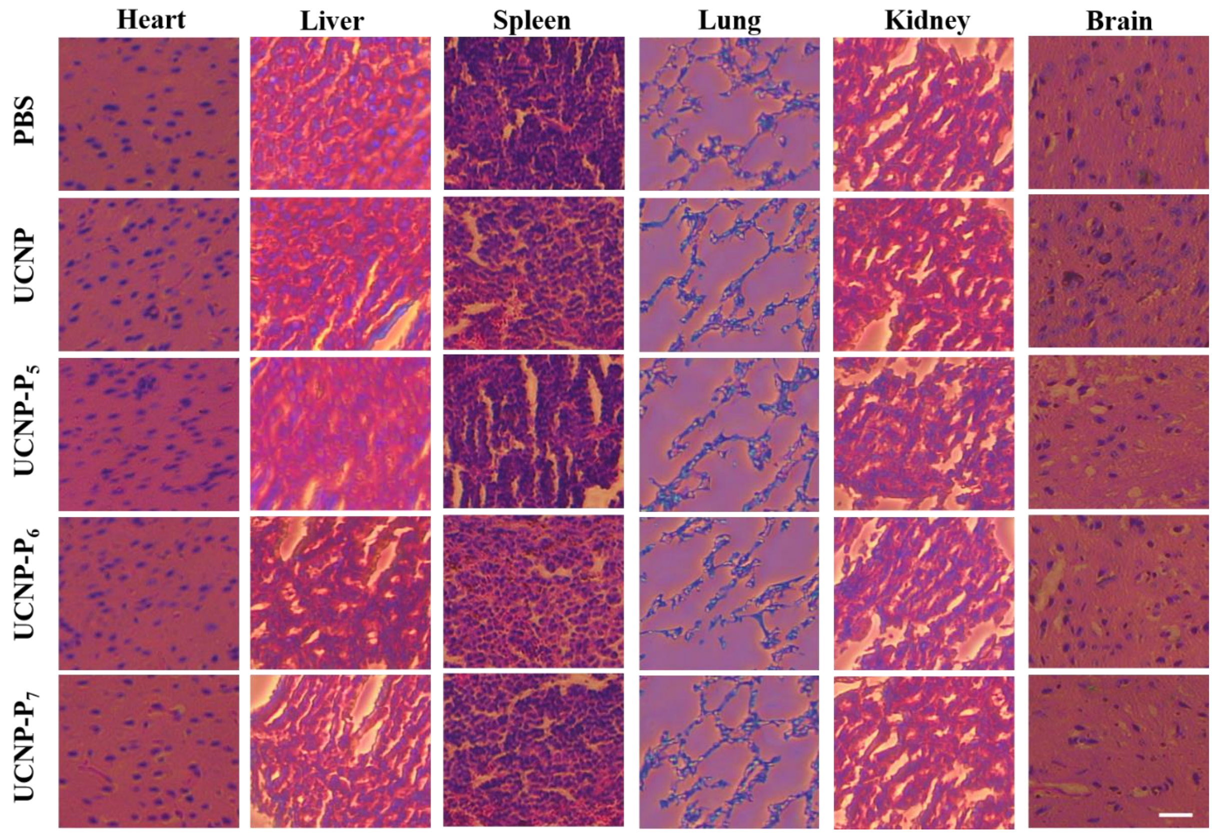

Figure S48. H&E staining of heart, liver, spleen, lung, kidney and brain from HeLa tumor-bearing mice after different treatments including PBS, UCNP, UCNP-P_5, UCNP-P_6 and UCNP-P_7. Scale bar = 100 µm.
